# Metabolic heterogeneity in clear cell renal cell carcinoma revealed by single-cell RNA sequencing and spatial transcriptomics

**DOI:** 10.1186/s12967-024-04848-x

**Published:** 2024-02-27

**Authors:** Guanwen Yang, Jiangting Cheng, Jiayi Xu, Chenyang Shen, Xuwei Lu, Chang He, Jiaqi Huang, Minke He, Jie Cheng, Hang Wang

**Affiliations:** 1 Department of Urology, Zhongshan Hospital, Fudan University, 180Th Fengling Rd, Xuhui District, Shanghai, 200032 China; 2 Department of Urology, Minhang Hospital, Fudan University, Shanghai, 201199 China; 3 Department of Urology, Xuhui Hospital, Fudan University, 966Th Huaihai Middle Rd, Xuhui District, Shanghai, 200031 China

**Keywords:** Clear cell renal cell carcinoma, Metabolic reprogramming, Single-cell RNA sequencing, Spatial transcriptome, Deep learning, Tumor microenvironment

## Abstract

**Background:**

Clear cell renal cell carcinoma is a prototypical tumor characterized by metabolic reprogramming, which extends beyond tumor cells to encompass diverse cell types within the tumor microenvironment. Nonetheless, current research on metabolic reprogramming in renal cell carcinoma mostly focuses on either tumor cells alone or conducts analyses of all cells within the tumor microenvironment as a mixture, thereby failing to precisely identify metabolic changes in different cell types within the tumor microenvironment.

**Methods:**

Gathering 9 major single-cell RNA sequencing databases of clear cell renal cell carcinoma, encompassing 195 samples. Spatial transcriptomics data were selected to conduct metabolic activity analysis with spatial localization. Developing scMet program to convert RNA-seq data into scRNA-seq data for downstream analysis.

**Results:**

Diverse cellular entities within the tumor microenvironment exhibit distinct infiltration preferences across varying histological grades and tissue origins. Higher-grade tumors manifest pronounced immunosuppressive traits. The identification of tumor cells in the RNA splicing state reveals an association between the enrichment of this particular cellular population and an unfavorable prognostic outcome. The energy metabolism of CD8^+^ T cells is pivotal not only for their cytotoxic effector functions but also as a marker of impending cellular exhaustion. Sphingolipid metabolism evinces a correlation with diverse macrophage-specific traits, particularly M2 polarization. The tumor epicenter is characterized by heightened metabolic activity, prominently marked by elevated tricarboxylic acid cycle and glycolysis while the pericapsular milieu showcases a conspicuous enrichment of attributes associated with vasculogenesis, inflammatory responses, and epithelial–mesenchymal transition. The scMet facilitates the transformation of RNA sequencing datasets sourced from TCGA into scRNA sequencing data, maintaining a substantial degree of correlation.

**Conclusions:**

The tumor microenvironment of clear cell renal cell carcinoma demonstrates significant metabolic heterogeneity across various cell types and spatial dimensions. scMet exhibits a notable capability to transform RNA sequencing data into scRNA sequencing data with a high degree of correlation.

**Supplementary Information:**

The online version contains supplementary material available at 10.1186/s12967-024-04848-x.

## Introduction

Renal cell carcinoma encompasses a diverse spectrum of diseases characterized by high invasiveness and marked heterogeneity in histological subtypes and mutational profiles [1]. Notably, clear cell renal cell carcinoma (ccRCC) stands as the predominant pathological type, contributing to a staggering annual global mortality exceeding 400,000 and displaying an upward trend in incidence [2].

Central to ccRCC's pathogenesis is its hallmark metabolic reprogramming, a multifaceted phenomenon that extends beyond the tumor cells and encompasses intricate interactions with the stromal and immune components within the tumor microenvironment [3–5]. Prior studies have revealed extensive alterations in central carbon metabolism, one-carbon metabolism, and antioxidant responses within the tumor tissue of ccRCC [6, 7]. These variations encompass glutathione, cysteine, and methionine metabolism [6], as well as metabolites in fatty acid oxidation [7, 8] and the degradation of branched-chain amino acids [9], which are associated with tumor metastasis and progression. The pentose phosphate pathway has been linked to sunitinib resistance [10], while L-2-hydroxyglutarate has been identified to promote tumor angiogenesis [11]. These findings span a diverse array of metabolites and tumor phenotypes.

However, these studies primarily involve the isolation of tumor cells either individually or by analyzing the entire tumor tissue block [12], relying on the assumption that “metabolic reprogramming mainly originates from tumor cells”. These studies emphasize the metabolic reprogramming of tumor cells while overlooking the influence of other cell types within the tumor microenvironment and their equally manifested metabolic reprogramming features. Recent research indicates that various cell types beyond tumor cells exhibit metabolic heterogeneity characteristics in multiple cancers, such as ovarian cancer [13], colorectal cancer [14], and hepatocellular carcinoma [15]. For instance, CD8 + T cells display suppressed glycolytic activity, leading to immune incompetence [16], and macrophages exhibit insufficient glucose supply, resembling M2 polarization features [17, 18]. However, the metabolic reprogramming and associated biological characteristics of the tumor microenvironment in ccRCC remain unclear.

Single-cell sequencing is a powerful tool for analyzing the gene expression profiles of individual cells and is instrumental in investigating metabolic heterogeneity in the tumor microenvironment [19]. However, it lacks the capability to retain spatial positional information. The advent of spatial transcriptomics addresses this limitation [20]. Therefore, we constructed a comprehensive, high-resolution single-cell atlas of ccRCC to explore the metabolic reprogramming in the tumor microenvironment. Additionally, we incorporated spatial transcriptomics to further dissect metabolic heterogeneity in spatial locations. We observed metabolic reprogramming phenomena and related biological features in various cell types, including the heterogeneity of energy metabolism in the evolution of CD8 + T cells and the strong correlation between sphingolipid metabolism and M2 polarization in macrophages. We categorized the spatial distribution of metabolites into discrete, peripheral, and central types. Furthermore, we developed the scMet package, a Python program capable of deconvolving RNA-seq data into single-cell RNA-seq data, aiming to facilitate the clinical application of single-cell sequencing.

## Materials and methods

### Single-cell sequencing data acquisition, integration, and processing

We retrieved raw expression data matrices, corresponding sample information, and patient data from nine previously published ccRCC studies [21–29]. Due to data incompleteness in the second part of the Braun Da et al. [28] database, only the first half of the data was included, and sample-related information can be found in Additional file 8: Table S1. Cell quality was controlled through two dimensions: the number of genes detected within a cell and the proportion of mitochondria-related genes. Specifically, cells with fewer than 200 detected genes or a mitochondria gene proportion exceeding 10% were filtered out. Genes were also cleaned, retaining those expressed in three or more cells. To eliminate doublets, Scrublet [30] was employed to detect doublets in each database, setting the expected doublet rate to 0.05. Cells with a predicted doubletScore greater than 0.3 were considered doublets and removed.

All data were merged using the Merge function, retaining genes that were present in all databases. Subsequently, 13,975 genes were selected for further analysis. The merged dataset was processed using the Seurat [31] package with default parameters, including standardization, normalization, and identification of highly variable genes. Given the presence of batch effects among multiple databases, we employed the Harmony [32] package to remove batches, using “Sample” or “Database” as grouping variables. To prevent overcorrection, we set the parameters sigma = 0.2, lambda = 1, early_stop = TRUE, and max_iter = 10. Notably, the grouping variable "Database" yielded more effective batch removal compared to "Sample." Therefore, "Database" was chosen for subsequent batch removal analysis.

The “harmony” components generated by Harmony [32] were utilized as substitutes for the principal components (PCs) in the subsequent analysis. Within the Seurat package [31], the FindNeighbors function was employed with the parameters set to reduction = “harmony” and dims = 1:15. This analysis was conducted on each cell to obtain the similarity distances between cells. FindClusters was then applied for further clustering analysis of the dimension-reduced cells. The parameters were set to resolution = 0.5 (applicable to the analysis of all cells) or resolution = 0.35 (applicable to further dimension reduction clustering of cell subtypes). To generate the final UMAP plot, the RunUMAP function was employed with parameters consistent with those of FindNeighbors.

### Obtaining, processing, and visualizing spatial transcriptomics data for regions of interest

The spatial transcriptomics data were sourced from the study conducted by Meylan et al. [33], accessed via the accession ID GSE175540, and generated using gene expression microarrays. In the quality control phase, genes expressed in fewer than 5 spots, spots expressing fewer than 300 features, and spots with a mitochondrial gene proportion exceeding 30% were excluded. Normalization was performed using the SCTransform method from Seurat [31] with default parameters. Cell type abundance was estimated using MCPcounter [34]. For unsupervised analysis, Independent Component Analysis (ICA) was applied using Seurat's RunICA, FindNeighbors, FindClusters, and RunUMAP functions. This analysis pipeline was executed across multiple spatial slides.

The center of circular envelope regions was used as the center of the plot. The plotting formulas for the two slices are as follows:

$${\text{Slice}}\,\,1:{\text{Distance}} = ({\text{column}} + 0.25*{\text{row}} - 70)/132)^{2} + (({\text{row}} - 31)/78*0.65)^{2}$$

$${\text{Slice}}\,2:{\text{Distance}} = ({\text{column}} - 67)/128*0.8)^{2} + (({\text{row}} - 42)/78)^{2}$$

The distance represents the spatial relationship with the center.

## Results

### Profiling tumor microenvironment heterogeneity in clear cell renal cell carcinoma reveals diverse cellular infiltration patterns

**Fig. 1:**
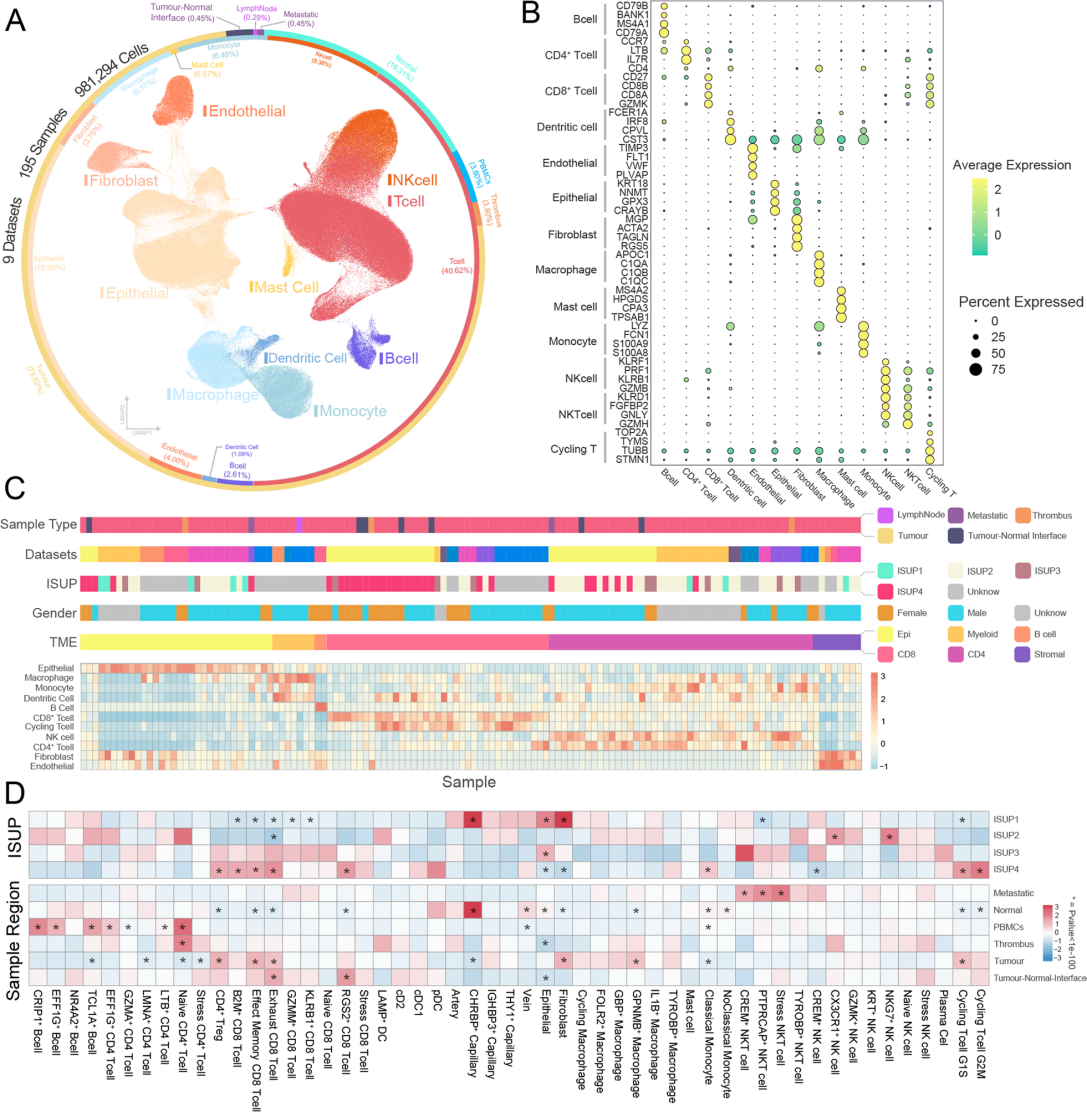
High-resolution single-cell atlas of clear cell renal cell carcinoma. **A** UMAP visualization of ccRCC, with the inner ring representing cell type proportions and the outer ring indicating tissue origin proportions. **B** Dot plot illustrating marker gene expression for major cell types. **C** Heatmap displaying sample information, tumor microenvironment classification groups, and cell infiltration enrichment levels for tumor samples. **D** Heatmap showcasing cell infiltration enrichment levels in the tumor microenvironment across different ISUP histological grades and sampling sites. ISUP: International Society of Urological Pathology; UMAP: Uniform Manifold Approximation and Projection

Metabolic reprogramming stands as a defining characteristic across the spectrum of malignancies [4]. Notably, among the ten prevalent malignancies, clear cell renal cell carcinoma(ccRCC) and hepatocellular carcinoma exhibit the most pronounced disparities in metabolic gene expression between tumors and corresponding normal tissues (Additional file 1: Fig. S1A, B). We curated publicly available single-cell RNA sequencing data for clear cell renal cell carcinoma (ccRCC) and performed quality control and filtering. In the end, we retained 195 samples from 76 patients for high-resolution mapping of the tumor microenvironment, including transcriptomic data from 981,294 cells for subsequent analysis (Fig. 1A and Additional file 8: Table S1).

Utilizing established lineage markers, we identified distinct cellular subpopulations: B cells (2.61%, CD79A/CD79B/BANK1), T cells (40.62%, CD3E/CD3G/CD45), Dendritic cells (1.08%, CST3/IRF8/CPVL), Endothelial cells (4.00%, TIMP3/FLT1/VWF), Fibroblasts (3.75%, ACTA2/TAGLN/RGS5), Macrophages (6.57%, C1QA/C1QB/APOC1), Mast cells (0.97%, MS4A2/HPGDS/CPA3), Monocytes (6.46%, FCN1/S100A8/S100A9), NK cells (8.38%, KLRF1/PRF1/KLRB1), and epithelial cells (25.93%, KRT18/NNMT/GPX3) (Fig. 1A, B, Additional file 1: Fig. S1C, and Additional file 9: Table S2). We performed non-negative matrix [35] factorization based on the proportions of these cell types within the tumor microenvironment (TME) for each sample, categorizing them into six groups: Epithelial^hi^ TME, Myeloid^hi^ TME, B cell^hi^ TME, CD8^+^ T cell^hi^ TME, CD4^+^ T cell^hi^ TME, and Stromal^hi^ TME) (Fig. 1C). Remarkably, a conspicuous enrichment of high-grade tumors was evident in the CD8^+^ T cell^hi^ TME, while the Epithelial^hi^ TME and Stromal^hi^ TME were primarily associated with low-grade tumors, underscoring the heterogeneous distribution of cells across distinct tumor grades. Following the normalization of cell numbers, we evaluated the degree of enrichment of each cell type within different histological grades and sampling sites (Fig. 1D). In addition to corroborating prior research findings of the enrichment of exhaust T cells and CD4^+^ regulatory T cells within high-grade tumors [28], our study reveals a distinct landscape. Within high-grade ISUP tissue samples, we observe enrichment of effect memory T cells and cycling T cells, while epithelial and fibroblast populations are notably sparse. Conversely, within low-grade tumors, we identify an abundance of fibroblasts, epithelial cells, and CHRBP^+^ cap endothelial cells. Notably, the infiltration of various CD8^+^ T cell subtypes, including exhaust T cells, effect memory T cells, and GZMM^+^ CD8^+^ T cells, is diminished in low-grade tumors. These observed phenomena collectively suggest a pronounced immunosuppressive milieu within high-grade ccRCC tissues underscoring a scarcity of tumor and stromal cells in this context, accentuating the complex interplay between immune responses, tumor microenvironment, and histological grades in ccRCC. While stress T cells have recently been implicated in immunotherapy responses [36], our analysis did not reveal significant differences in their presence across various tissue types. Moreover, significant disparities in cellular infiltration were observed between different sampling sites of the tumor tissues (Fig. 1D). Within metastatic lesions, we discern a notable enrichment of diverse subtypes of NKT cells. Intriguingly, the patterns of cellular infiltration observed in normal tissues mirror those identified in low-grade ISUP tissues. These patterns are distinguished by a robust presence of fibroblasts, epithelial cells, and CHRBP^+^ cap endothelial cells, coupled with a conspicuous paucity of CD4 regulatory T cells and CD8^+^ exhaust T cells. Naive T cells and B cells are predominantly localized within the peripheral blood. In the tumor-normal boundary, CD8^+^ T cells, especially exhaust T cells, exhibited significant infiltration, whereas epithelial infiltration was less pronounced.

### Unraveling the complexities of glycolysis and its diverse biological impacts on tumorigenesis

**Fig. 2:**
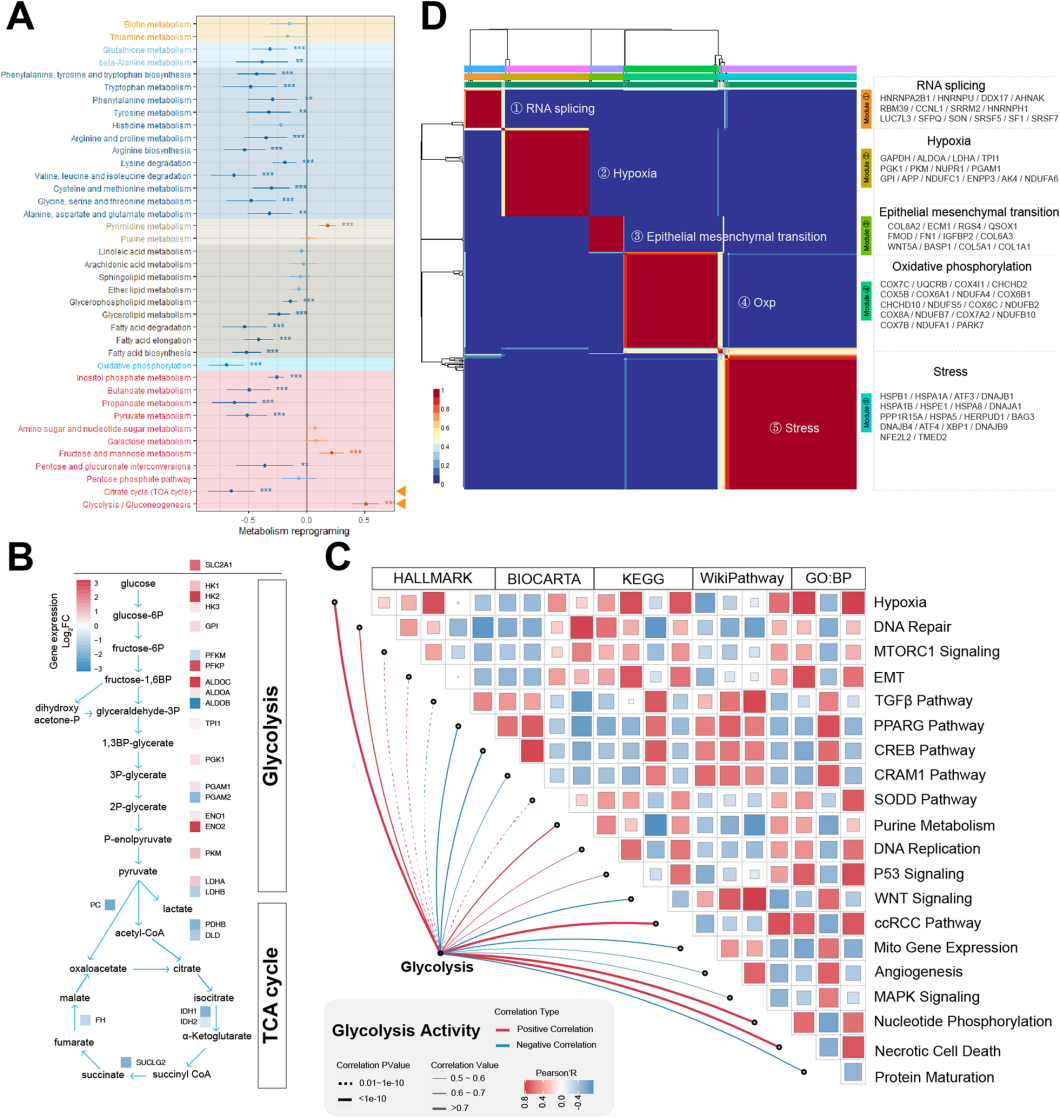
Evident enhanced glycolytic activity in tumor cells of clear cell renal cell carcinoma. **A** Forest plot depicting the differential activity of multiple metabolic pathways between tumor cells and normal epithelial cells, with different background colors representing distinct metabolic categories. **B** Differential expression of genes related to Glycolysis and Tricarboxylic Acid (TCA) Cycle between tumor cells and normal epithelial cells, highlighted in red for high expression in tumor cells and blue for high expression in normal epithelial cells. **C** Correlation heatmap showing the association between glycolytic activity in tumor cells and various biological functions. **D** Correlation heatmap of different states of tumor cells, where red indicates high correlation and blue indicates low correlation. EMT: Epithelial–Mesenchymal Transition; Oxp: Oxidative Phosphorylation

Identification of copy number variations (CNVs) using InferCNVpy [37] was employed to determine tumor cells (Additional file 2: Fig. S2A), followed by a differential analysis of metabolic pathway activities between tumor and normal cells (Fig. 2A). The findings revealed a pervasive attenuation in the functionality of numerous metabolic pathways within tumor cells, encompassing amino acid metabolism, carbohydrate metabolism, and a portion of fatty acid metabolism. Conversely, a distinct manifestation of the Warburg effect was evident [38], whereby tumor cells prominently displayed heightened glycolytic activity, concomitant with a notable reduction in both oxidative phosphorylation and tricarboxylic acid cycle engagement. This metabolic reprogramming underscores the dynamic adaptations occurring in tumor cell energy metabolism and further underscores the multifaceted nature of tumor bioenergetics. The escalated proliferative propensity of tumor cells engendered a heightened requisition of nucleotides, culminating in an augmented pyrimidine metabolism. Among the pivotal pathways of metabolic reprogramming, glycolysis exhibited an upsurge in gene expression across the majority of its constituents, except for ALDOB. This metabolic shift was concurrently characterized by the downregulation of crucial genes integral to the tricarboxylic acid cycle. Within the glycolysis pathway, genes such as HK2, PFKP, and PKM displayed elevated expression, thereby emerging as prospective targets warranting exploration for restraining tumor cell glycolytic activity (Fig. 2B and Additional file 2: Fig. S2B–D).

Antecedent investigations have established a connection between heightened glycolysis and augmented DNA damage repair mechanisms within neoplastic cells [39]. Within our research, we unearthed a correlation between DNA repair processes and glycolytic activity in tumor cells of ccRCC. Moreover, we elucidated a positive association between the activity of DNA replication, cellular proliferation, and purine metabolism with glycolytic activity (Fig. 2C and Additional file 2: Fig. S2E). These revelations posit that escalated glycolytic activity could potentially drive an augmentation in cellular proliferation. The mTORC1 pathway emerged as a pivotal orchestrator, orchestrating this phenomenon by engendering and governing two pivotal transcription factors, namely HIF1 and Myc, which in turn fostered the glycolytic cascade [40]. Furthermore, a discernible correlation between mTORC1 signaling and the amplitude of glycolytic activity was unveiled. Equally noteworthy, signaling pathways, including the p53, MAPK, WNT, and PPARG pathways, also exhibited discernible correlations with glycolytic activity (Fig. 2C and Additional file 2: Fig. S2E). Prior investigations have underscored the pivotal role of glycolysis in fostering tumor metastasis. This phenomenon is elucidated by the relocalization of glycolytic enzymes from the cytoplasm to the nucleus, thereby augmenting the expression of critical transcription factors intricately linked with the EMT [41]. Our findings unveiled a discernible positive correlation between the activity of epithelial–mesenchymal transition within tumor cells and glycolytic activity. The SODD pathway, recognized as the silencer of death domains [42], and acknowledged for its heightened expression in thwarting TNF-induced cell death and restraining NF-kB activation, exhibited a notable positive correlation with glycolytic activity. However, the precise underlying mechanism necessitates additional comprehensive validation. Furthermore, the activity of the necrotic cell death pathway, intricately linked with cell demise, displayed a concurrent positive association with glycolysis.

Intra-tumor heterogeneity stands out as a salient characteristic of tumors, exerting a pivotal influence on drug responsiveness [43]. Past investigations have unveiled the presence of intra-tumor heterogeneity in ccRCC, encompassing the manifestation of hypoxia, stress, and EMT-associated patterns [24, 44, 45]. In our study, we aimed to depict a more comprehensive tumor landscape by eliminating batch effects between samples (Fig. 2D and Additional file 2: Fig. S2F). We initiated an intra-sample clustering of tumor cells to derive average expression profiles representing various states of these cells. Subsequently, employing non-negative matrix factorization [35] across all tumor samples, we effectively classified tumor cells into five distinct states, each assigned a categorical nomenclature: RNA splicing, Hypoxia, EMT, OXP, and Stress (Fig. 2D). Furthermore, beyond the previously recognized Hypoxia/EMT/Stress states, we identified a distinct cluster of tumor cells exhibiting RNA splicing activity. We identified a cluster of tumor cells in an RNA splicing state and observed that patients with enrichment of tumor cells in this cluster exhibited poorer prognoses (Additional file 2: Fig. S2G). Analogously, clusters of tumor cells characterized by intensified EMT and OXP-related traits exhibited unfavorable prognostic implications (Additional file 2: Fig. S2I, J). Intriguingly, an elevated expression of genes pertinent to the Hypoxia state within tumor cells correlated with a more favorable prognosis (Additional file 2: Fig. S2H). The enrichment of tumor cells in the Stress state did not exhibit a discernible relationship with patient prognosis, underscoring a comparatively weaker association with overall survival (Additional file 2: Fig. S2K).

### Metabolic dynamics and glycolysis interplay in CD8^+^ T cell immune responses

**Fig. 3:**
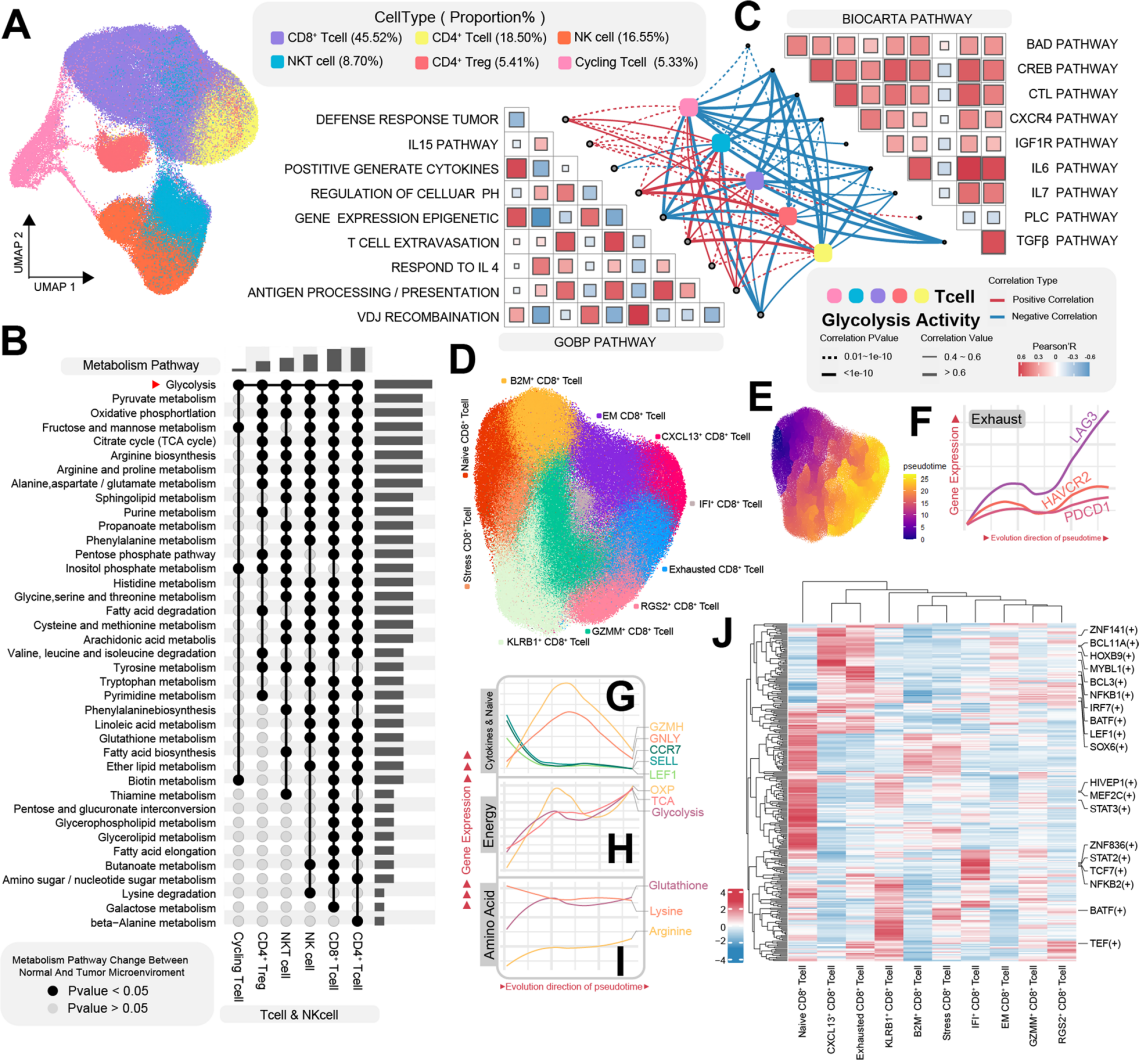
Metabolic heterogeneity and dynamics of T cells in the tumor microenvironment. **A** UMAP plot of T cells and NK cells, color-coded by cell type. **B** Differences in Metabolic Activity of Various T Cell Subtypes Between Tumor Microenvironment and Normal Tissue Microenvironment, Represented by Solid and Hollow Circles Signifying P-values < 0.05 and > 0.05, respectively. **C** Correlation of glycolytic activity in various T cell types with multiple biological functions. **D** UMAP plot of CD8^+^ T cells, color-coded by cell subtype. **E** UMAP plot of CD8^+^ T cells, color-coded by inferred pseudotime trajectory position, ranging from black (trajectory start) to yellow (trajectory end). **F** Exhaustion marker gene expression in T cells along the inferred pseudotime trajectory. **G** Line plots depicting the expression of cytotoxicity and naive T cell marker genes along the inferred pseudotime trajectory. **H** Line plot showing dynamic changes in energy metabolism activity along the inferred pseudotime trajectory. **I** Line plot illustrating dynamic changes in amino acid metabolism activity along the inferred pseudotime trajectory. **J** Heatmap of transcription factor activity in CD8^+^ T cells, with red indicating high activity and blue indicating low activity. UMAP: Uniform Manifold Approximation and Projection

T cells, a highly enriched cell population within the tumor microenvironment of ccRCC, experience transcriptional, translational, and epigenetic modifications driven by both tumor cell metabolites and diverse immune signals [46]. These changes lead to metabolic reprogramming, allowing T cells to adapt to the intricate and dynamic tumor microenvironment. To gain deeper insights into the metabolic deviations within tumor-infiltrating T cells, we isolated T cells and NK cells for subsequent UMAP clustering analysis. Using well-established lineage markers, we further classified T cells into distinct subtypes: CD8^+^ T cells (45.52%, CD8A/CD8B/GZMM), CD4^+^ T cells (18.50%, IL7R/CD4), NKT cells (8.70%, CD3E/GZMH), CD4^+^ Tregs (5.41%, FOXP3/TIGIT/CTLA4), Cycling T cells (5.41%, MKI67/TOP2A), along with NK cells (16.55%, NKG7/GNLY/KLRD1) (Fig. 3A, Additional file 3: Fig. S3A and Additional file 9: Table S2).

Conducting differential analysis of metabolic pathway activity within T cells and NK cells, grouped by normal and tumor tissues as criteria, revealed CD4^+^ T cells and CD8^+^ T cells as the cell subsets undergoing the most pronounced metabolic reprogramming (Fig. 3B). The metabolic pathway activity between these two lineages exhibited marked differences, reflecting their distinct functional roles. In contrast, cycling T cells exhibited relatively subtle metabolic distinctions, potentially attributed to their specialized biological functions. Among the various metabolic pathways examined, those related to energy metabolism demonstrated the most discrepancies. Glycolysis exhibited divergent activity patterns across all six major cell types, while pyruvate metabolism and oxidative phosphorylation displayed altered activity in all cell types except for cycling T cells. Consequently, building upon these findings, we proceeded to investigate the relationship between T cell glycolysis activity and specific biological behaviors (Fig. 3C). Our analysis unveiled a negative correlation between glycolysis and several BIOCARTA gene sets, including the IL6 Pathway, IL7 Pathway, CTL (Mediated immune response against target cells) Pathway, BAD (Pro-apoptotic molecule) Pathway, PLC (Phospholipase C Signaling Pathway) Pathway, and CXCR4 Pathway. Conversely, the correlation between T cell glycolysis activity and GOBP gene sets presented varying trends. For instance, T cell extravasation, antigen processing and presentation, defense response to tumors, regulation of cellular pH, and the IL15 Pathway exhibited positive correlations with glycolysis activity. In contrast, VDJ Recombination and cytokine generation showed negative correlations with T cell glycolysis activity.

In pursuit of a deeper comprehension of the metabolic transformations occurring within CD8^+^ T cells during cellular evolution, we conducted further sub clustering of CD8^+^ T cells and categorized them based on their distinctive expressed genes. These subclusters of CD8^+^ T cells were designated as follows: Naive CD8^+^ T cells (CCR7/LEF1/SELL), Stress CD8^+^ T cells (JUN/FOS/HSP Related Gene), B2M^+^ CD8^+^ T cells (B2M), Effect Memory CD8^+^ T cells (CD74/CD27/NKG7), CXCL13^+^ CD8^+^ T cells (CXCL13/XCL1/XCL2), IFI^+^ CD8^+^ T cells (IFI Related Genes), Exhausted CD8^+^ T cells (PDCD1/LAG3/HAVCR2), RGS2^+^ CD8^+^ T cells (RGS2/DNAJB1), GZMM^+^ CD8^+^ T cells (GZMM/GZMK), and KLRB1^+^ CD8^+^ T cells (KLRB1/ANXA1) (Fig. 3D and Additional file 9: Table S2). Pseudotime analysis was performed using Monocle3 [47], with naive CD8^+^ T cells designated as the starting point of the cellular evolutionary trajectory (Fig. 3E and Additional file 9: Fig. S3E). Markers associated with exhaustion and inhibitory checkpoints (LAG3/HAVCR2/PDCD1) exhibited a gradual increase along the trajectory. Conversely, cytokine markers (GZMH, GNLY) displayed an expression pattern characterized by an initial rise followed by a decline. Markers indicative of a naive state (SELL, LEF1, CCR7) showed a gradual decrease during the evolution in pseudotime (Fig. 3G). This observation suggests a well-fitted trajectory progressing from a naive state through a cytokine-enriched phase to an exhausted state. Employing enrichment analysis of metabolism-related genes for cells at distinct states along the trajectory, we uncovered intriguing patterns. Energy metabolism-related pathways demonstrated an initial surge followed by a subsequent decline and then a secondary increase in activity (Fig. 3H). The first peak of energy demand aligned with the period of heightened cell-killing effectiveness, while the second peak coincided with the timeframe marked by the enrichment of Exhausted CD8^+^ T cells. This observation suggests that robust energy metabolism is vital not only for CD8^+^ T cells to execute their effector functions [48] but also characterizes CD8^+^ T cell exhaustion. Amino acid metabolism exhibited distinct trends, with arginine metabolism experiencing a gradual decrease and glutathione and arginine metabolism showing a gradual increase (Fig. 3I).

In a subsequent step, we conducted SCENIC TFs (Transcription Factors) analysis specifically centered on CD8^+^ T cells, aiming to uncover the principal regulatory factors governing their differentiation and exhaustion processes. (Fig. 3J). The outcomes of this analysis unveiled discernible activities of transcription factors across distinct cell subclusters. Notably, Naive CD8^+^ T cells exhibited the highest abundance of highly active transcription factors. Among these, MEF2C(+) and STAT3(+) stood out as transcription factors with elevated expression levels. In contrast, GZMM^+^ CD8^+^ T cells and Effect Memory CD8^+^ T cells displayed fewer instances of highly active transcription factors. Exhausted CD8^+^ T cells notably exhibited substantially augmented activity in BCL3(+) and NFKB1(+) transcription factors. Additionally, CXCL13^+^ CD8^+^ T cells showcased elevated expression of MYBL1(+). These transcription factors may wield crucial roles in orchestrating T cell differentiation and could potentially serve as therapeutic targets.

### Metabolic heterogeneity shapes macrophage functions in tumor microenvironment

**Fig. 4:**
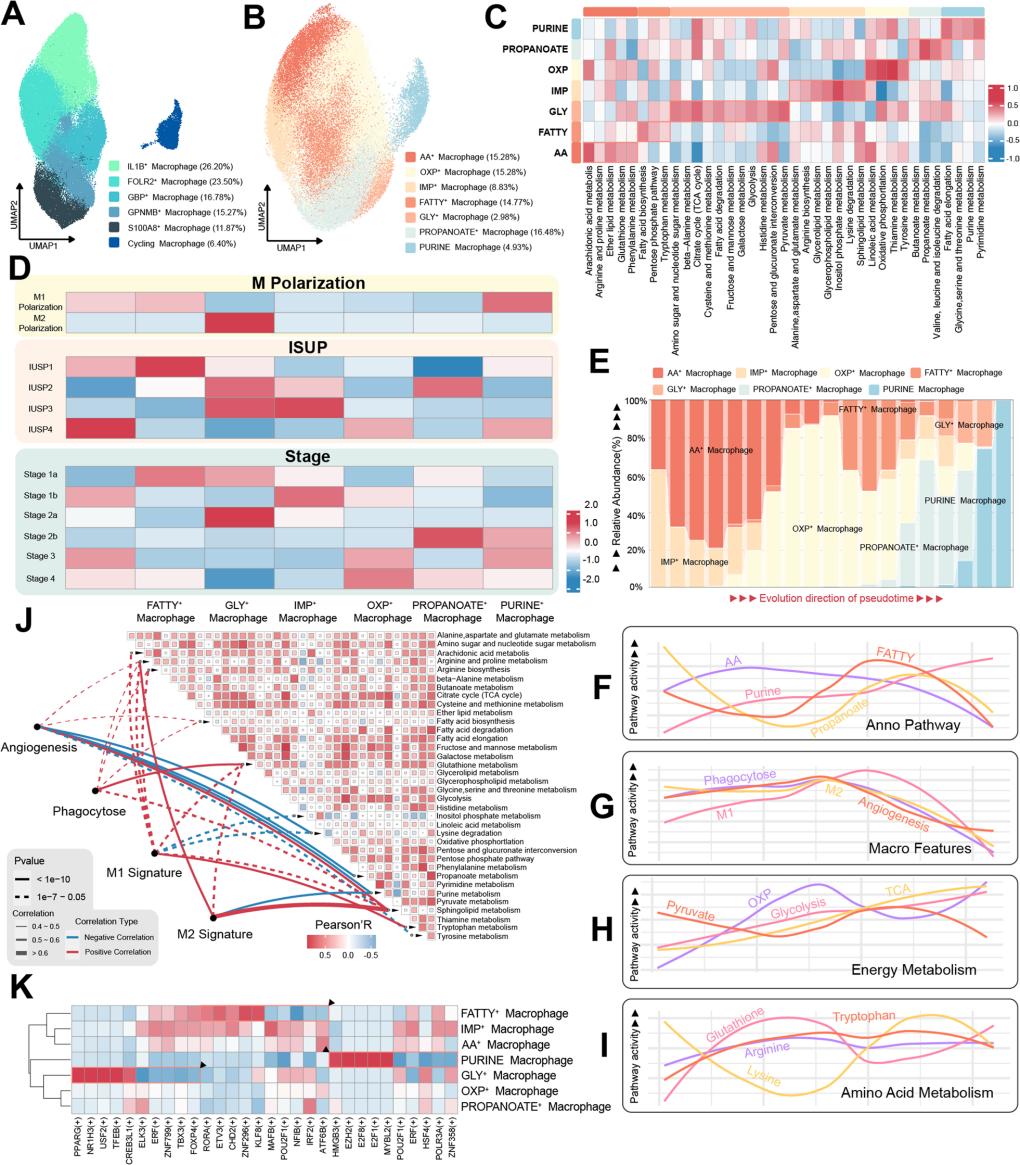
Macrophage metabolism and its highly correlated biological significance. **A** UMAP plot of macrophages, color-coded by cell subtype. **B** UMAP plot of macrophages, color-coded by metabolic subtype. **C** Metabolic activity heatmap of macrophages across different metabolic subtypes. **D** Infiltration enrichment heatmap of macrophage subtypes in different polarizations, ISUP histological grades, and tumor grades. **E** Dynamic changes in enrichment levels of different macrophage subtypes along the inferred pseudotime trajectory. **F** Dynamic changes in annotated metabolic pathway activity of macrophage metabolic subtypes along the inferred pseudotime trajectory. **G** Dynamic changes in macrophage feature activity along the inferred pseudotime trajectory. **H** Dynamic changes in energy metabolism activity of macrophages along the inferred pseudotime trajectory. **I** Dynamic changes in amino acid metabolism activity of macrophages along the inferred pseudotime trajectory. **J** Correlation heatmap between macrophage feature activity and various metabolic pathway activities. **K** Transcription factor activity heatmap of macrophages, where red indicates high activity and blue indicates low activity. IMP: Inositol phosphate metabolism; AA: Arachidonic acid metabolism; OXP: Oxidative phosphorylation; GLY: Glycolysis; FATTY: Fatty acid metabolism; ISUP: International Society of Urological Pathology.UMAP: Uniform Manifold Approximation and Projection

The metabolic heterogeneity in the liver tumor microenvironment has been well-documented [49], but its manifestation in ccRCC has not been elucidated yet. To further analyze the metabolic differences in macrophages, we isolated macrophages (C1QA/C1QB/C1QC), performed UMAP clustering, and named the clusters based on highly expressed genes. The clusters were designated as IL1B^+^ Macrophage (26.20%, IL1B/EREG/AREG), FOLR2^+^ Macrophage (23.50%, FOLR2/EGR1/MAF), GBP^+^ Macrophage (16.78%, GBP1/GBP4), GPNMB^+^ Macrophage (15.27%, GPNMB/APOC1/CTSD), S100A8^+^ Macrophage (11.87%, S100A8/FTL), and Cycling Macrophage (6.40%, MKI67/TOP2A) (Fig. 4A, Additional file 4: Fig. S4A and Additional file 9: Table S2). Within each distinct subcluster of macrophages, we conducted GSVA focusing on metabolic pathway activity. This examination unveiled significant metabolic disparities across various subclusters of macrophages (Additional file 4: Fig. S4B).

To probe into the functional implications of diverse metabolic states in macrophages, we initiated a re-clustering process grounded in genes linked to metabolism. This analysis gave rise to the identification of seven distinct subclasses: AA^+^ Macrophage (15.28%, exhibited increased arachidonic acid and glutathione metabolism pathway activity), OXP^+^ Macrophage (15.28%, showed enhanced oxidative phosphorylation and tryptophan metabolism pathway activity), IMP^+^ Macrophage (8.83%, displayed elevated inositol phosphate metabolism pathway activity), FATTY^+^ Macrophage (14.77%, demonstrated increased fatty acid synthesis metabolism pathway activity), GLY^+^ Macrophage (2.98%, presented heightened glycolysis activity), PROPANOATE^+^ Macrophage (16.48%, displayed enhanced propionate metabolism activity), and PURINE^+^ Macrophage (4.93%, exhibited high expression of purine and pyrimidine metabolism) (Fig. 4B, C). Through an analysis of the enrichment levels of distinct metabolic state macrophages across varying polarization statuses, ISUP histological grades, and tumor grades (Fig. 4D), we unveiled that GLY^+^ Macrophages primarily exhibited enrichment within M2-polarized macrophages, while AA^+^ Macrophages, FATTY^+^ Macrophages, and PURINE^+^ Macrophages emerged as notably enriched within M1-polarized macrophages. In the context of ISUP histological grades and tumor stages, FATTY^+^ Macrophages are predominantly heir present in low-grade tumors, in contrast to AA^+^ Macrophages which exhibited enrichment in high-grade tumors.

To simulate the dynamic course of metabolic evolution in macrophages, we executed a trajectory simulation grounded in the expression profiles of metabolic genes (Additional file 4: Fig. S4C, D). Our investigation unveiled a distinctive metamorphosis of macrophage metabolic states, transitioning from an amalgam of IMP^+^ Macrophages and AA^+^ Macrophages at the early phase of cellular evolution, advancing to OXP^+^ Macrophages and FATTY^+^ Macrophages during the intermediary stage, and ultimately culminating in PROPANOATE^+^ Macrophages and PURINE^+^ Macrophages at the final juncture of the trajectory (Fig. 4E). The dynamic changes in the activity of metabolic pathways used to name the cell clusters align with the corresponding enrichment of cell clusters in the pseudotime trajectory (Fig. 4F). For instance, the peak activity of the FATTY metabolic pathway coincides with the highest enrichment of FATTY Macrophage cells in the pseudotime trajectory. The examination of characteristic gene set expressions in certain macrophages revealed that attributes related to M1 polarization, M2 polarization, phagocytosis, and angiogenesis initially demonstrated an elevation in activity, which was subsequently followed by a decline (Fig. 4G). These attributes reached their peak levels at the time when OXP^+^ Macrophages were most abundant, suggesting a potential interplay between OXP^+^ Macrophages and the modulation of these cellular functions. It is worth mentioning that OXP^+^ Macrophages are also enriched in high-grade tumors. Based on these findings, we hypothesize that OXP^+^ Macrophages may play a crucial role as the predominant macrophage subtype in the tumor microenvironment. Regarding energy metabolism, the activity trends of oxidative phosphorylation, tricarboxylic acid cycle, and glycolysis exhibited incremental augmentation, contrasting the gradual decline in pyruvate metabolism (Fig. 4H). Distinct trends were observed in the metabolism of various amino acids, with arginine and proline metabolism showing no pronounced alterations (Fig. 4I). Glutathione metabolism displayed an initial rise followed by a subsequent decline along the pseudotime trajectory. Intriguingly, analogous to the metabolic dynamics in CD8^+^ T cells, we similarly noted that lysine metabolism exhibited a converse trend compared to glutathione metabolism, characterized by an initial decline followed by an eventual ascent.

Drawing on the aforementioned discoveries, we analyzed the relationship between macrophage characteristics and metabolic pathway activity. Sphingolipid metabolism exhibited a notable positive correlation with all four macrophage traits, with a particularly pronounced link to M2 polarization (Fig. 4J). Tryptophan metabolism displayed its highest correlation with M1 polarization, while glutathione metabolism was most closely associated with phagocytic activity. Our examination of macrophage transcription factors highlighted distinctive activities across various metabolic states (Fig. 4K). For instance, GLY^+^ Macrophages exhibited significant elevations in the activities of transcription factors PPARG( +), NR1H3( +), USF2( +), and TFEB( +), with PPARG( +) having established ties to Th9 glycolysis [50]. Similarly, PURINE^+^ Macrophages displayed heightened activities in HMGB3( +), EZH2( +), E2F8( +), and E2F1( +) transcription factors, with HMGB3( +) implicated in DNA repair and EZH2( +) functioning as a pivotal chromatin regulator [51, 52].

### ENPP2 as a potential prognostic marker and tumor-originating endothelial cell indicator

**Fig. 5:**
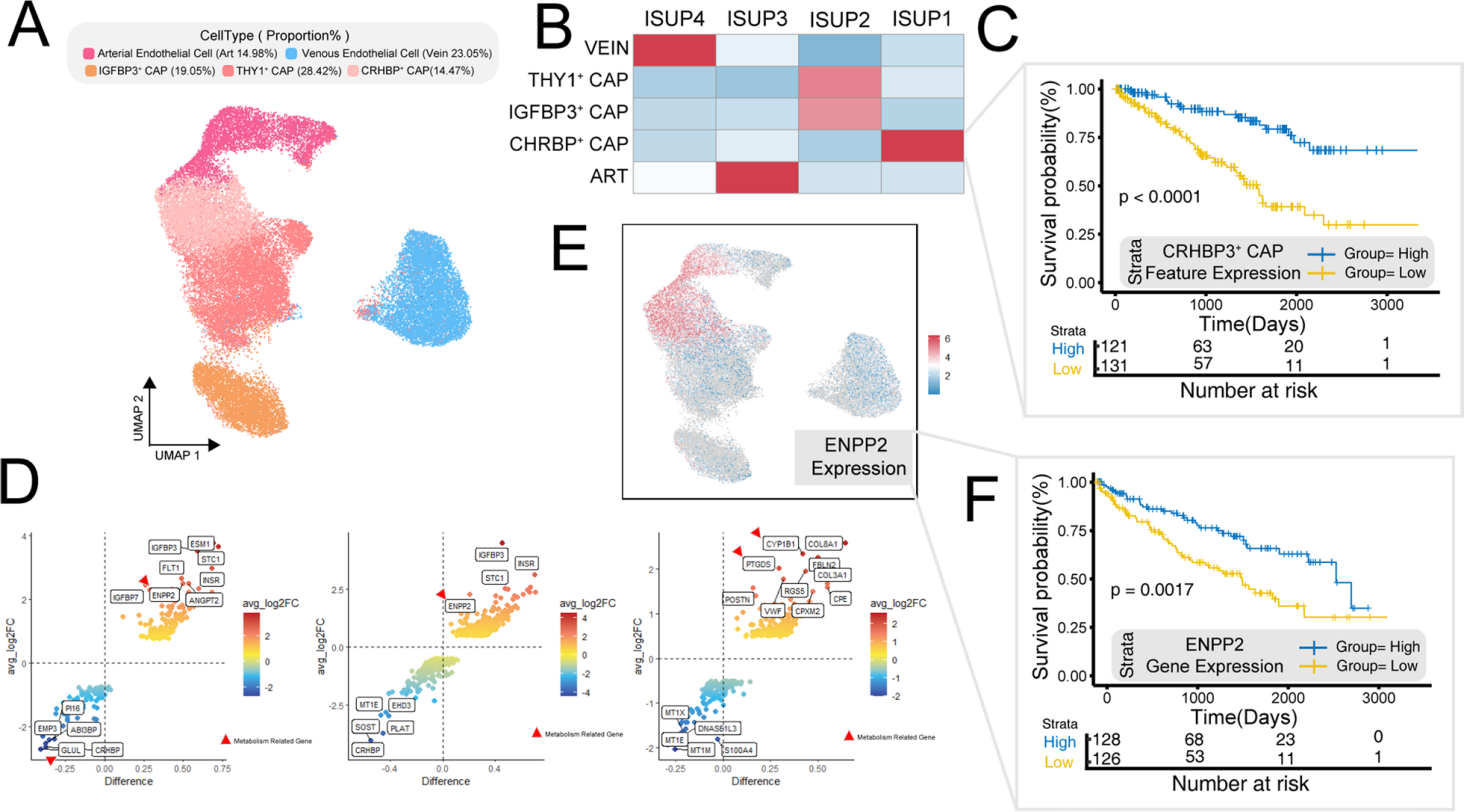
Endothelial cell expression heterogeneity. **A** UMAP plot of endothelial cells, color-coded by cell subtypes. **B** Enrichment heatmap of different endothelial cell subtypes across various ISUP grades of tumor tissue. **C** Impact of CRHBP^+^ CAP endothelial cell enrichment on patient prognosis. **D** Differential genes among cell subtypes derived from tumor and normal sources: Left-Arterial endothelial cells; Middle-Capillary endothelial cells; Right-Venous endothelial cells. **E** UMAP plot of endothelial cells, color-coded by expression of ENPP2 gene. **F** Patient prognosis stratified by ENPP2 expression levels. ISUP: International Society of Urological Pathology.UMAP: Uniform Manifold Approximation and Projection

Previous studies have indicated the metabolic plasticity of endothelial cells during pathological angiogenesis [53]. Here, we isolated endothelial cells and classified them into distinct subtypes, namely venous endothelial cell (23.05%, VWF/CLU/VCAM1), arterial endothelial cell (14.98%, SOX6/GLUL/PI16), IGFBP3^+^ capillary endothelial cell (19.05%, IGFBP3/ENPP2/ANGPT2), THY1^+^ capillary endothelial cell (28.42%, THY1/COL4A1/CA4), and CHRBP3^+^ capillary endothelial cell (14.47%, CHRBP3/SOST/IGFBP5) (Fig. 5A, Additional file 5: Fig. S5A, B and Additional file 9: Table S2).

In various histological grades, we noted a predominant enrichment of CHRBP3^+^ capillary endothelial cells in low-grade tumors (Fig. 5B). Consequently, we leveraged the enrichment level of the CHRBP3^+^ capillary endothelial cell-specific gene set as a basis for grouping in survival prognosis analysis. This analysis unveiled that patients with elevated expression of this gene set within their tumors displayed markedly improved overall survival (Pvalue < 0.0001) (Fig. 5C). This observation implies a potential association between CHRBP3^+^ capillary endothelial cells and a favorable prognosis. We identified an enrichment of venous endothelial cells in high-grade tumors (Fig. 5B). However, when the extent of enrichment of the venous endothelial cell gene signature was employed for survival analysis grouping, no significant prognostic disparities emerged among the patients (Additional file 5: Fig. S5E). This suggests that the prevalent vascular endothelial cells in high-grade tumors may predominantly exhibit venous characteristics, yet this phenotypic manifestation does not seem to correlate with patient prognosis. Nevertheless, additional validation is imperative to affirm the reliability and implications of these findings.

We performed differential analysis on venous endothelial cells, arterial endothelial cells, and capillary endothelial cells from different sources and found that arterial endothelial cells and capillary endothelial cells exhibited strikingly similar differences (Fig. 5D). ENPP2 was identified as a common differentially expressed gene in both cell types and is also a metabolism-related gene (Fig. 5D). Interestingly, ENPP2 was primarily expressed in endothelial cells and not in other cell types (Fig. 5E and Additional file 5: Fig. S5D). Survival prognosis analysis based on ENPP2 expression showed that patients with higher expression of this endothelial marker had better prognostic outcomes (Fig. 5F). Therefore, ENPP2 can serve as a specific biomarker for tumor-derived endothelial cells and a prognostic indicator. In contrast, the differentially expressed genes in venous endothelial cells were more unique in tumor tissues. In tumor tissues, venous endothelial cells exhibited higher expression of metabolism-related genes such as CYP1B1 and PTGDS, the heightened expression of these genes was associated with poorer prognostic outcomes (Additional file 5: Fig. S5F–H).

### Mapping spatial metabolic activity in ccRCC microenvironment

**Fig. 6:**
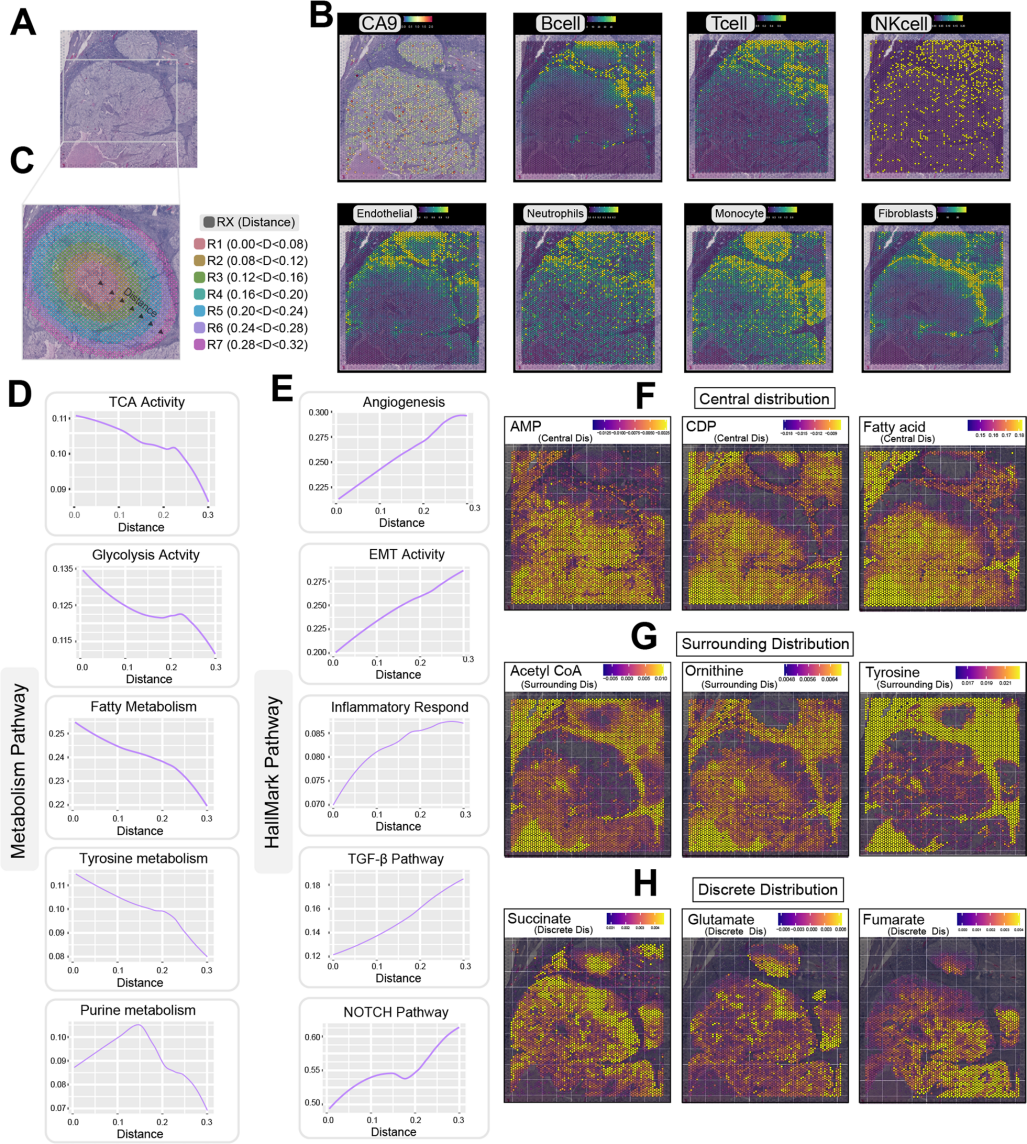
Slice 1: heterogeneity in spatial metabolic activity. **A** Pathological section of spatial transcriptomics. **B** Approximate distribution of various cell types. **C** Partitioning of spatial transcriptomics data, colored by distance from the tumor center. **D** Correlation of metabolic pathways (glycolysis, TCA cycle, fatty acid metabolism, tyrosine, and purine metabolism) with distance from the tumor center. **E** Correlation of biological pathway activities (EMT, angiogenesis, inflammation, TGF-beta, and NOTCH) with distance from the tumor center. **F** Heatmap of balance flux for AMP, CDP, and fatty acids in the spatial context. **G** Heatmap of balance flux for acetyl CoA, ornithine, and tyrosine in the spatial context. **H** Heatmap of balance flux for succinate, glutamate, and fumarate in the spatial context. EMT—Epithelial–Mesenchymal Transition

Spatial transcriptomics preserves transcriptomic data within its spatial context, facilitating the analysis of metabolic pathway activity in localized regions. Before this study, metabolic activity within the spatial dimension of ccRCC had not been characterized. To establish the link between metabolic activity and spatial information, we utilized two spatial transcriptomic datasets featuring nearly circular tumor pathological regions (Fig. 6A and Additional file 6: Fig. S6A). The central point of the tumor was taken as a simulated tumor core. Employing MCPcounter [34], we calculated the estimated distribution of various cell types across the tissue sections (Fig. 6B and Additional file 6: Fig. S6B).

The spatial distribution of the tumor within the envelope was segmented into multiple regions based on their distance from the central point, and these regions were appropriately labeled (Fig. 6C and Additional file 6: Fig. S6C). In both of the selected datasets, a consistent negative correlation was observed between the activities of metabolic processes such as the citric acid cycle, glycolysis, fatty acid metabolism, and tyrosine metabolism, and the distance from the tumor center (Fig. 6D and Additional file 6: Fig. S6D). This observation indicates a prevailing heightened metabolic state at the tumor center, while the energy metabolism activity diminishes in the proximity of the tumor envelope. Interestingly, purine metabolism, often linked to cell proliferation, demonstrated its most pronounced activity not at the tumor center but rather at the midsection between the tumor center and the envelope (Fig. 6D and Additional file 6: Fig. S6D). The presence of features associated with angiogenesis exhibited an ascending trend as the distance from the tumor center increased (Fig. 6E and Additional file 6: Fig. S6E). This suggests that regions rich in blood vessels are more frequently found in the peripheral areas around the tumor, whereas the tumor core has a lower density of blood vessels. This scarcity of blood vessels in the tumor center could potentially contribute to the establishment of hypoxic conditions within the tumor microenvironment. Similarly, gene sets that displayed heightened expression near the tumor envelope were characterized by activities linked to processes like epithelial–mesenchymal transition and inflammatory responses. This highlights that tumor cells located close to the envelope have a greater inclination toward migration and invasive behavior. Moreover, the activities of the TGF-β and NOTCH pathways exhibited a positive correlation with increasing distance from the tumor center. This observation hints at the potential involvement of these signaling pathways in regulating spatial heterogeneity within the tumor microenvironment.

By employing scFEA [54], we computed the spatial equilibrium distribution of diverse metabolites within the context of spatial transcriptomic data. In both instances of spatial transcriptomic datasets, we discerned noteworthy variances in the spatial distribution of specific metabolites. AMP, CDP, and fatty acid exhibited a pronounced proclivity for enrichment within the tumor region, characterized by a “Central” distribution pattern (Fig. 6F and Additional file 6: Fig. S6F). Conversely, acetyl-coA, ornithine, and tyrosine demonstrated a heightened propensity for enrichment in the proximate periphery of the tumor envelope, manifesting a distinctive “Surrounding” distribution profile (Fig. 6G and Additional file 6: Fig. S6G). Intriguingly, despite the enrichment of succinate, glutamate, and fumarate within the tumor region, their distribution did not exhibit a continuous trajectory, instead adopting a discernible “Discrete” disposition (Fig. 6H and Additional file 6: Fig. S6H).

### scMet: a deep learning-based approach for accelerating clinical application of scRNA-seq in cancer

**Fig. 7:**
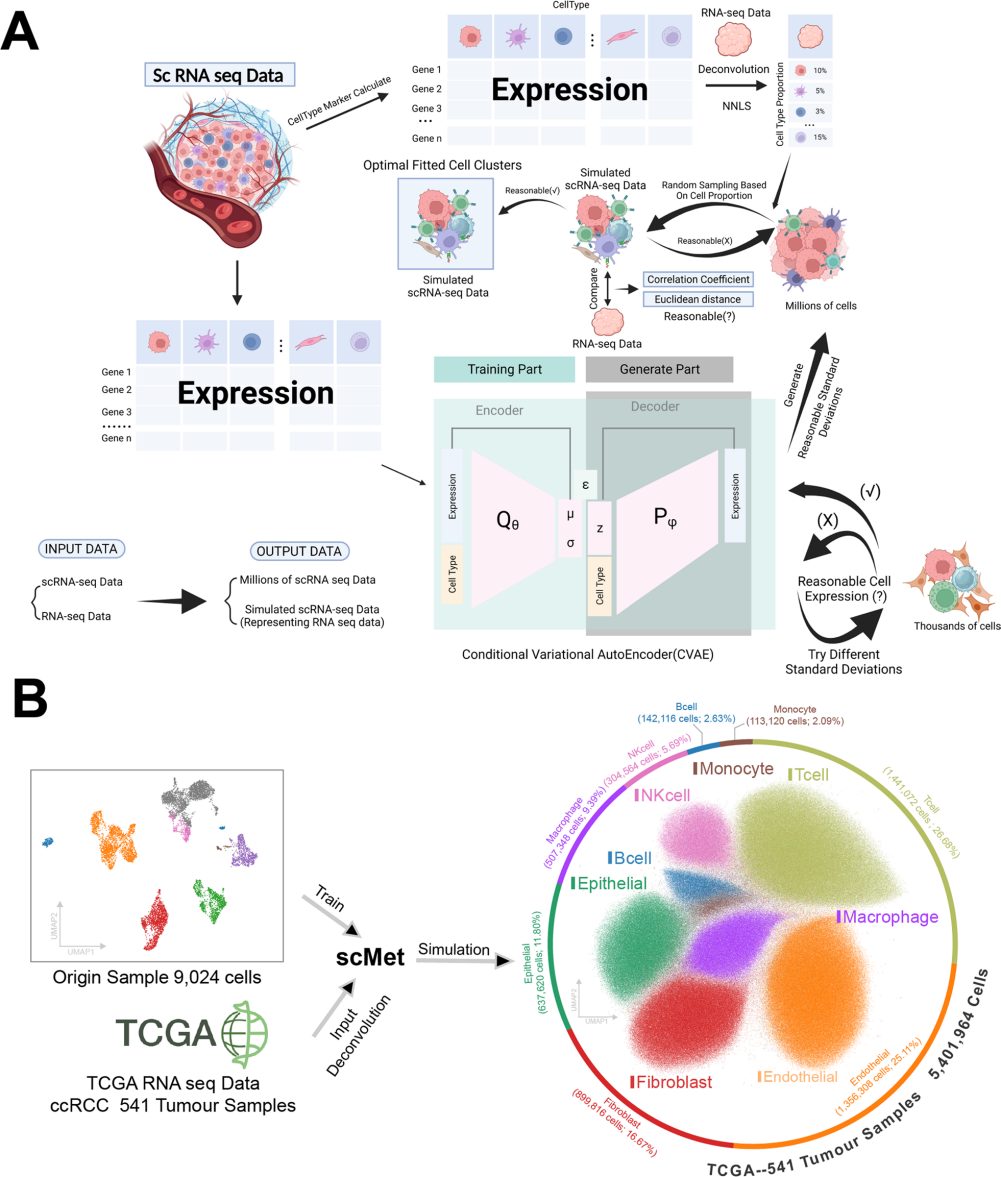
scMet workflow and conversion of TCGA RNA-seq data. **A** Design flowchart of the scMet program. **B** Workflow illustrating the conversion of TCGA RNA-seq data into scRNA-seq data using scMet

scRNA-seq empowers the analysis of tissue-level RNA expression with unprecedented single-cell precision, making it a pivotal tool for both patient stratification and the unraveling of tumor mechanisms [55]. Nevertheless, there exist formidable impediments to the clinical integration of scRNA-seq, encompassing its considerable expenses and stringent sample prerequisites [55]. In response, we have introduced scMet, an innovative solution harnessed on a deep learning platform (conditional variational auto-encoder), with the primary goal of transmuting bulk RNA sequencing data into scRNA-seq-like profiles (Fig. 7A, Methods). This endeavor is poised to streamline the clinical deployment of scRNA-seq by mitigating these challenges.

To achieve this, we leveraged the sustainable data generation capability of a conditional variational auto-encoder (CVAE) model. We used scRNA-seq data as input for training the model, iteratively reducing the loss (Additional file 7: Fig. S7A). Through continuous learning iterations, the model generated data with the most realistic simulation effect, demonstrating the ability to generate a large volume of scRNA seq data. In the subsequent steps, we selected different standard deviations to generate small-scale scRNA-seq data with varying degrees of dispersion. This generated data was then applied to the routine single-cell sequencing pipeline, enabling the generation of UMAP plots to assess the credibility of the generated data. Ultimately, we identified the most suitable standard deviation and employed the well-trained model to generate millions of single cell expression data for fitting RNA-seq data. Furthermore, to enhance the efficiency of the fitting process, we utilized scRNA-seq data to compute the expression of cell-type specific marker genes. This information was then employed to perform deconvolution on the RNA-seq data, yielding approximated proportions of different cell types (Additional file 7: Fig. S7B, C). We explored various numbers of cell-type specific marker genes and found that they consistently maintained an acceptable level of consistency with the original samples. Ultimately, we cyclically selected cell clusters from the generated millions of single-cell expression data based on the computed proportions of different cell types. This selection process was guided by correlation analysis with original RNA-seq data and Euclidean distance calculations (Additional file 7: Fig. S7D, E). The chosen set of scRNA-seq data exhibited the highest similarity to the gene expression characteristics of the original RNA-seq samples.

The TCGA database comprises RNA-seq data from 541 ccRCC samples, accompanied by comprehensive patient prognostic and gene mutation information. In this study, we utilized scMet to transform RNA-seq data from TCGA into a scRNA-seq data matrix containing 5,401,964 cells and 3000 metabolism-related genes, resulting in over 15 billion RNA expression values (Fig. 7B). Following the completion of the fitting process, the scRNA-seq data matrix was subjected to downstream analysis using Scanpy. The generated UMAP plots demonstrated that the scRNA-seq data, intended to substitute for RNA-seq data in downstream analysis, exhibited robust cell clustering performance. In the scenario of using a small sample for training the CVAE model, represented by a dataset containing merely 1,278 cells, scMet continued to perform well in converting eight TCGA RNA-seq datasets into scRNA-seq data (Additional file 7: Fig. S7F). Furthermore, these individual samples were effectively integrated, showing minimal batch effects.

## Discussion

Cell infiltration within the tumor microenvironment plays a pivotal role in tumor progression and significantly influences the efficacy of immune checkpoint therapy [56]. Salcher S conducted a study that categorized the tumor microenvironment of lung adenocarcinoma into distinct types, characterized by prevailing B cells, T cells, myeloid cells, and tumor cells [57]. This investigation examined the distribution of diverse pathways, including the Wnt pathway, across various tumor microenvironments. Similarly, in our research, we classified the tumor microenvironment in ccRCC, identifying six distinct types. Our analysis revealed that tumor microenvironments enriched with CD8^+^ T cells are predominantly found in high-grade tumors, corroborating the findings of Braun DA [28]. This observation aligns with the immunosuppressive microenvironment commonly observed in high-grade tumors. Moreover, our study introduced a novel revelation—the enrichment of epithelial cells and fibroblasts in low-grade tumors. This discovery represents a previously undocumented phenomenon and underscores the complexity of the tumor microenvironment in ccRCC.

Liang's work proposed a mechanism wherein the glycolytic enzyme PGK1 relocates to the cell nucleus, binding to the core promoter region of CDH1, thereby repressing E-cadherin expression and inducing EMT [58]. Our research aligns with this proposition, as we have uncovered a correlation between glycolysis and EMT in ccRCC. Furthermore, we have identified a connection between glycolysis and the TGF-β pathway, mirroring observations made in other contexts such as pulmonary arterial hypertension and non-small cell lung carcinoma [59, 60]. In a broader context, our findings suggest potential correlations between glycolysis and processes such as cell proliferation, apoptosis, and specific signaling pathways. Nevertheless, it's important to note that many of these relationships are still unverified, and the underlying mechanisms within ccRCC remain undisclosed. Consequently, further experimental validation is warranted to elucidate these associations and mechanisms fully.

Prior investigations have extensively delved into the intricacies of tumor heterogeneity within ccRCC and proposed a variety of comparable meta-programs, such as Stress, Hypoxia, EMT, and Antigen Presentation [24, 44, 45]. Our research not only substantiates these established understandings but also uncovers a previously unexplored meta-program involving RNA splicing within tumor cells and accentuate the enrichment of a tumor subtype associated with RNA splicing alterations, which is linked to less favorable prognoses. Dysregulated RNA splicing stands as a recognized hallmark of cancer [61]. Studies conducted by Chang et al. identified 16 enriched abnormal structural variations in ccRCC [62], while the work of Xiao L revealed the involvement of selective RNA splicing in cancer stem cells, consequently fostering tumorigenesis through a multitude of mechanisms [63]. This phenomenon leads to escalated cell proliferation, diminished apoptosis, heightened migration, and metastatic potential, increased resistance to chemotherapy, and evasion of immune surveillance.

Inhibition of metabolism, particularly glycolysis, can lead to T cell dysfunction [64]. Suppressing glycolysis not only impairs the biosynthetic capacity required for effector cell proliferation and function but also affects crucial energy-sensing and growth-regulatory signaling pathways essential for immune cell activation [65]. Among all metabolic pathways, glycolytic changes are most pronounced across different tissue microenvironments. IL-7, through STAT5-mediated AKT activation, promotes Glut1 transport and glucose uptake [15]. Interestingly, we observed a negative correlation between IL-7 and glycolytic activity. This could be due to the absence of GLUT1 in the glycolytic activity pathway gene set.

Kishore M proposed the substantial role of glycolysis in CD4 Treg migration [66]. Furthermore, glycolysis serves to enhance T cell cytotoxicity against tumor cells [67]. In alignment with this understanding, our research has unveiled correlations connecting T cell extravasation, tumor response, and antigen processing presentation with glycolytic activity. We have also made an unprecedented discovery—a negative correlation between VDJ rearrangement and glycolysis. Our metabolic analysis of T cells has uncovered dynamic shifts in T cell metabolism upon activation. Beyond the widely acknowledged heightened metabolic activity of effector T cells [67], our study has revealed a resurgence of elevated energy demands during T cell exhaustion. This novel insight casts fresh illumination on the intricate metabolic dynamics inherent to the evolution of T cells (see Additional file 10, 11).

Li S investigated the role of metabolism in shaping macrophage phenotypes within the hepatic microenvironment [49]. Their study highlighted purine metabolism as a defining characteristic of terminally differentiated macrophages. We observed that macrophages exhibiting enrichment in purine metabolism are particularly prevalent in high-grade tumors. This suggests that these macrophages have entered a terminal state marked by diminished macrophage traits. Conversely, OXP^+^ macrophages in high-grade tumors signify a state where macrophages play a predominant role, in line with the proposal by Wculek SK that oxidative phosphorylation regulates macrophage homeostatic activity across different tissues [68]. Sphingolipids, significant constituents of eukaryotic cell membranes, emerge as crucial lipids governing macrophage activities [69]. Constituting approximately a quarter of macrophage membrane lipids, sphingolipids play pivotal roles in phagocytosis, lysosomal stability, vesicle fusion, receptor-mediated chemotaxis, autophagy, and antigen presentation. Significantly, our findings establish a robust correlation between sphingolipid metabolism and various functional traits of macrophages. This positions sphingolipid metabolism as a promising target for the modulation of macrophage biological functions, potentially offering new avenues for therapeutic interventions.

ENPP2 has previously been proposed as a source of tumor endothelial cells in ccRCC [70]. We extended this finding by identifying ENPP2 as a marker for tumor artery and capillary-derived endothelial cells, rather than tumor venous endothelial cells. Additionally, we uncovered the prognostic significance of ENPP2. Rohlenova K depicted metabolic reprogramming during pathological endothelial cell generation using single-cell sequencing [53], offering crucial insights for developing anti-angiogenic therapies for tumors. Hence, investigating the metabolic dynamics occurring in the process of aberrant vessel formation in ccRCC holds further promise.

Liu YM revealed a phenomenon of elevated oxidative phosphorylation distribution along the leading edge of breast cancer [71]. We unveil, for the first time, spatially defined metabolic heterogeneity in ccRCC. We identify a high-energy metabolic state at the tumor center and heightened purine metabolism in the intermediate zone. Beyond the frequently mentioned epithelial–mesenchymal transition features enriched in the tumor-normal interface, we further discover elevated inflammatory response and angiogenesis characteristics surrounding the tumor envelope.

To overcome limitations in the clinical application of scRNA-seq and fully utilize RNA-seq data with comprehensive prognostic information, there have been similar approaches in the past. For instance, MuSiC2 [72] can analyze RNA-seq data, with a focus on accurate cell-type deconvolution. Similarly, BayesPrism [73] goes beyond cell type deconvolution to depict gene expression levels for each cell type. However, it still falls short of generating scRNA-seq data. Bulk2Space [74], on the other hand, primarily converts RNA-seq into spatially resolved single-cell data, with an emphasis on spatial redistribution. However, it lacks quality control for the generated data and subsequent fitting of RNA-seq, which affects data usability. We developed scMet, prioritizing the quality of generated scRNA-seq data and its correlation with RNA-seq. Our goal is to create scRNA-seq data that accurately represents RNA-seq and is suitable for downstream analysis. scMet was tested on the TCGA database and demonstrated promising results. The fitted generated data exhibit clear interpretability, addressing the need for high-quality scRNA-seq data conversion from RNA-seq.

## Conlusions

The tumor microenvironment of ccRCC demonstrates significant metabolic heterogeneity across various cell types and spatial dimensions. scMet exhibits a notable capability to transform RNA sequencing data into scRNA sequencing data with a high degree of correlation.

### Supplementary Information

**Additional file 1: Figure S1.** Evident metabolic reprogramming in renal cell carcinoma. **A** Differential Metabolic Gene Scores between Tumor and Corresponding Normal Samples in the ten common tumors from TCGA. **B** Heatmap depicting metabolic activity in tumor and normal samples of Clear Cell Renal Cell Carcinoma, with red indicating high activity and blue indicating low activity. **C** UMAP visualization of samples color-coded by data sources. KIRC: Kidney Renal Clear Cell Carcinoma; LIHC: Liver Hepatocellular Carcinoma; COAD: Colon Adenocarcinoma; BRCA: Breast Carcinoma; STAD: Stomach Adenocarcinoma; LUAD: Lung Adenocarcinoma; PRAD: Prostate Adenocarcinoma; UCEC: Uterine Corpus Endometrial Carcinoma; THCA: Thyroid Carcinoma; PAAD: Pancreatic Adenocarcinoma; SKCM: Skin Cutaneous Melanoma; BLCA: Bladder Urothelial Carcinoma; UMAP: Uniform Manifold Approximation and Projection.

**Additional file 2: Figure S2.** Correlation of tumor cell glycolysis with pathways and prognosis in clear cell renal cell carcinoma. **A** Copy number variation heatmap in epithelial cells. **B**–**D** Immunohistochemical staining images of PFKP/ENO2/PKM in adjacent normal tissue and clear cell renal cell carcinoma. **E** Scatter plots depicting the correlation of glycolytic activity with various biological features. **F** Stability plot of non-negative matrix factorization (NMF) clustering of tumor cells at different group numbers. **G**–**K** Survival analysis of tumor cells enriched in different groups based on RNA splicing, Hypoxia, Oxidative Phosphorylation (Oxp), Epithelial–Mesenchymal Transition (EMT), and Stress states. EMT: Epithelial–Mesenchymal Transition; Oxp: Oxidative Phosphorylation; NMF: Non-Negative Matrix Factorization.

**Additional file 3: Figure S3.** Metabolic dynamics of CD8+ T cells. **A** UMAP plot of T cells and NK cells, color-coded based on marker gene expression. **B**–**D** UMAP plots of CD4^+^ T cells, NK cells, and NKT cells, color-coded by cell subtype. **E** Dynamic changes in enrichment levels of different CD8^+^ T cell subtypes along the inferred pseudotime trajectory. UMAP: Uniform Manifold Approximation and Projection.

**Additional file 4: Figure S4.** Dynamic evolution of macrophage metabolism. **A** UMAP plot of macrophages, color-coded by expression of marker genes. **B** Metabolic activity heatmap of macrophage subtypes across different cell subtypes. **C** UMAP plot of macrophages used for pseudotime analysis, color-coded by expression of marker genes. **D** UMAP plot of macrophages used for pseudotime analysis, color-coded by inferred pseudotime trajectory points, where black leans towards the starting point and yellow leans towards the endpoint of the trajectory. UMAP: Uniform Manifold Approximation and Projection.

**Additional file 5: Figure S5.** Correlation of tumor-originated endothelial cell-specific markers with prognosis. **A** UMAP plot of endothelial cells, color-coded based on their tissue of origin. **B**, **C** UMAP plots of endothelial cells, color-coded by the expression levels of specific marker genes. Red denotes high expression, blue denotes low expression. **D** UMAP plot displaying ENPP2 gene expression across all cells. Red indicates high expression, blue indicates low expression. **E** Prognostic analysis stratified by the expression of characteristic genes in venous endothelial cells. **F**–**H** Prognostic analysis stratified by the expression of IGFBP3, CYP1B1, and PTGDS genes. UMAP: Uniform Manifold Approximation and Projection.

**Additional file 6: Figure S6.** Slice 2: Heterogeneity in spatial metabolic activity. **A** Pathological section of spatial transcriptomics. **B** Approximate distribution of various cell types. **C** Partitioning of spatial transcriptomics data, colored by distance from the tumor center. **D** Correlation of metabolic pathways (glycolysis, TCA cycle, fatty acid metabolism, tyrosine, and purine metabolism) with distance from the tumor center. **E** Correlation of biological pathway activities (EMT, angiogenesis, inflammation, TGF-beta, and NOTCH) with distance from the tumor center. **F** Heatmap of balance flux for AMP, CDP, and fatty acids in spatial context. **G** Heatmap of balance flux for acetyl CoA, ornithine, and tyrosine in spatial context. **H** Heatmap of balance flux for succinate, glutamate, and fumarate in spatial context. EMT : Epithelial–Mesenchymal Transition.

**Additional file 7: Figure S7.** Figures for scMet program evaluation. **A** Correlation between the number of training iterations for the Conditional Variational Auto-Encoder (CVAE) model and the corresponding training loss and validation loss. **B** Correlation between the number of cell-type specific markers used for deconvolution of RNA sequencing data and the accuracy of computational results (Left), and correlation between the number of cell-type specific markers used for deconvolution of RNA sequencing data and computational time (Right). **C** Bar plot representing the cell type proportions obtained after deconvolution of TCGA RNA-seq data. **D** Line graph illustrating the gene expression correlation between the best-fitted scRNA-seq data and the original RNA-seq data. **E** Scatter plot depicting the correlation between gene expression of TCGA-CJ-5684-01A-11R-1541-07 RNA-seq data and the best-fitted scRNA-seq data. **F** Workflow illustrating the utilization of small sample scRNA-seq data to convert eight TCGA RNA-seq datasets into scRNA-seq data using scMet. CVAE: Conditional Variational Auto-Encoder.

**Additional file 8. Table S1.** Sample details extracted from the integrated single-cell databases utilized in the study.

**Additional file 9. Table S2.** Compilation of marker genes associated with identified cell subtypes.

**Additional file 10. Table S3.** Listing of gene names encompassed in the feature gene set employed in the study.

**Additional file 11. Supplementary Materials And Methods.** Expansion of the methodology section, details on software packages, and the operating platform used in the study.

## Data Availability

The single-cell RNA sequencing data by Chow J is available on the GEO database under Accession number GSE202374. Processed data from Saout JR’s single-cell RNA sequencing study can be accessed via the GEO portal with accession number GSE224630. Yu Z’s single-cell RNA sequencing data is accessible in the Gene Expression Omnibus datasets under accession number GSE207493. Li R’s single-cell RNA sequencing data can be downloaded as h5ad objects from Mendeley Data at https://doi.org/10.17632/g67bkbnhhg.1. Kim MC’s single-cell sequencing data has been deposited as GSE121638. Zhang Y’s single-cell RNA sequencing data is deposited in the National Center for Biotechnology Information Gene Expression Omnibus under Accession number GSE159115. Obradovic A’s single-cell sequencing data is available at https://github.com/Aleksobrad/single-cell-rcc-pipeline. Krishna C's single-cell sequencing data can be found at https://www.ncbi.nlm.nih.gov/sra/PRJNA705464. Braun DA’s data can be accessed through the Single Cell Portal at https://singlecell.broadinstitute.org/single_cell/study/SCP1288/tumor-and-immune-reprogramming-during-immunotherapy-in-advanced-renal-cell-carcinoma#study-summary. The spatial transcriptomics data of ccRCC is available on the Gene Expression Omnibus (GEO) database under the Accession number GSE175540. RNA-seq data for TCGA ccRCC and relevant clinical data are obtainable from https://www.cancer.gov/ccg/research/genome-sequencing/tcga. The immunohistochemistry images of clear cell renal cell carcinoma (ccRCC) can be found on https://www.proteinatlas.org/.The code for scFEA can be obtained from https://github.com/changwn/scFEA. The code for scMet and R code for calculating cell infiltration enrichment can be obtained from https://github.com/gwyang6/scMet.

## Abbreviations

ccRCC: Clear cell renal cell carcinoma
NMF: Non-negative matrix factorization
ISUP: International Society of Urological Pathology
KIRC: Kidney renal clear cell carcinoma
LIHC: Liver Hepatocellular Carcinoma
COAD: Colon Adenocarcinoma
BRCA: Breast Carcinoma
STAD: Stomach Adenocarcinoma
LUAD: Lung Adenocarcinoma
PRAD: Prostate Adenocarcinoma
UCEC: Uterine Corpus Endometrial Carcinoma
THCA: Thyroid Carcinoma
PAAD: Pancreatic Adenocarcinoma
SKCM: Skin Cutaneous Melanoma
BLCA: Bladder Urothelial Carcinoma
UMAP: Uniform Manifold Approximation and Projection
EMT: Epithelial–mesenchymal transition
IMP: Inositol phosphate metabolism
AA: Arachidonic acid metabolism
OXP: Oxidative phosphorylation
GLY: Glycolysis
FATTY: Fatty acid metabolism
CVAE: Conditional Variational Auto-Encoder
scRNA seq: Single-cell RNA sequencing
PCA: Principal Component Analysis
CNV: Copy Number Variation
GSVA: Gene Set Variation Analysis
TFs: Transcription Factors
