# Supplementary figures and images for: Metabolic heterogeneity in clear cell renal cell carcinoma revealed by single-cell RNA sequencing and spatial transcriptomics
Source: J Transl Med. 2024 Feb 27;22:210. doi: 10.1186/s12967-024-04848-x (PMC10900752; doi:10.1186/s12967-024-04848-x)

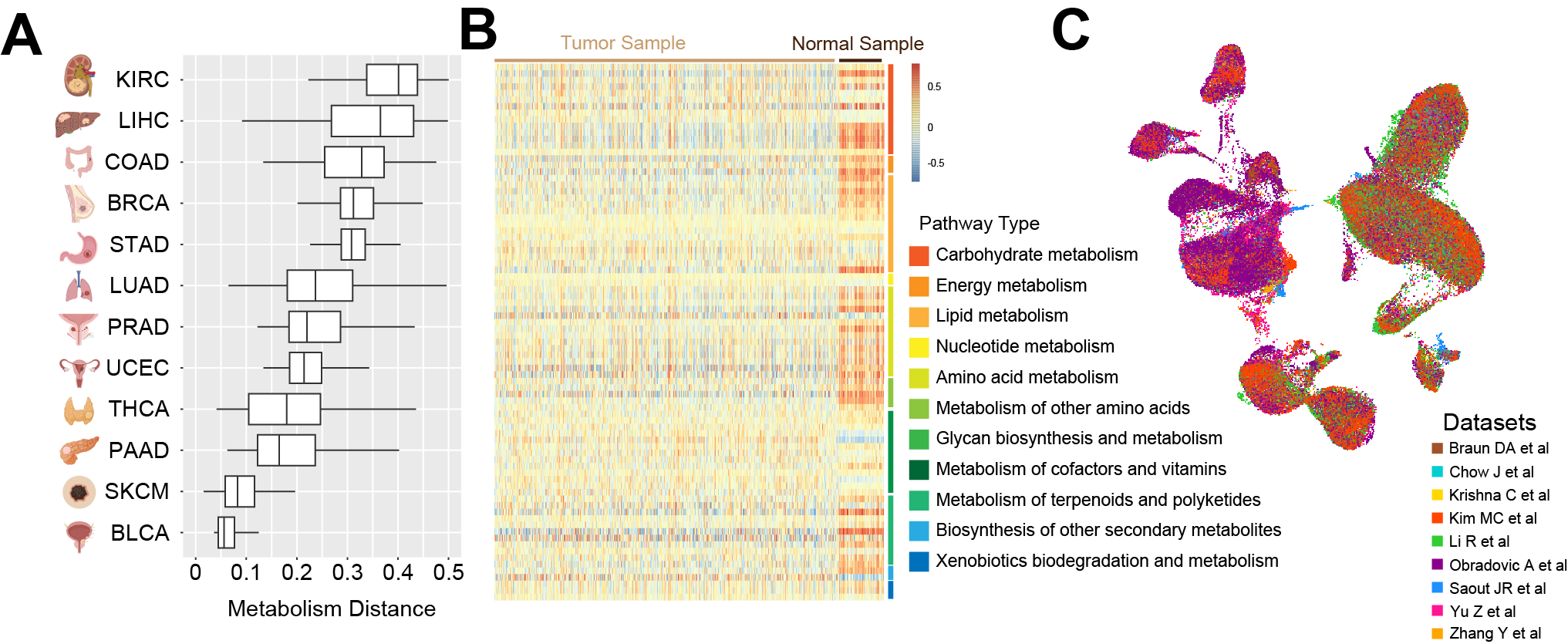

Supplement: Supplementary file 1 — Additional file 1: Figure S1. Evident metabolic reprogramming in renal cell carcinoma. A Differential Metabolic Gene Scores between Tumor and Corresponding Normal Samples in the ten common tumors from TCGA. B Heatmap depicting metabolic activity in tumor and normal samples of Clear Cell Renal Cell Carcinoma, with red indicating high activity and blue indicating low activity. C UMAP visualization of samples color-coded by data sources. KIRC: Kidney Renal Clear Cell Carcinoma; LIHC: Liver Hepatocellular Carcinoma; COAD: Colon Adenocarcinoma; BRCA: Breast Carcinoma; STAD: Stomach Adenocarcinoma; LUAD: Lung Adenocarcinoma; PRAD: Prostate Adenocarcinoma; UCEC: Uterine Corpus Endometrial Carcinoma; THCA: Thyroid Carcinoma; PAAD: Pancreatic Adenocarcinoma; SKCM: Skin Cutaneous Melanoma; BLCA: Bladder Urothelial Carcinoma; UMAP: Uniform Manifold Approximation and Projection. [file 12967_2024_4848_MOESM1_ESM.tif]

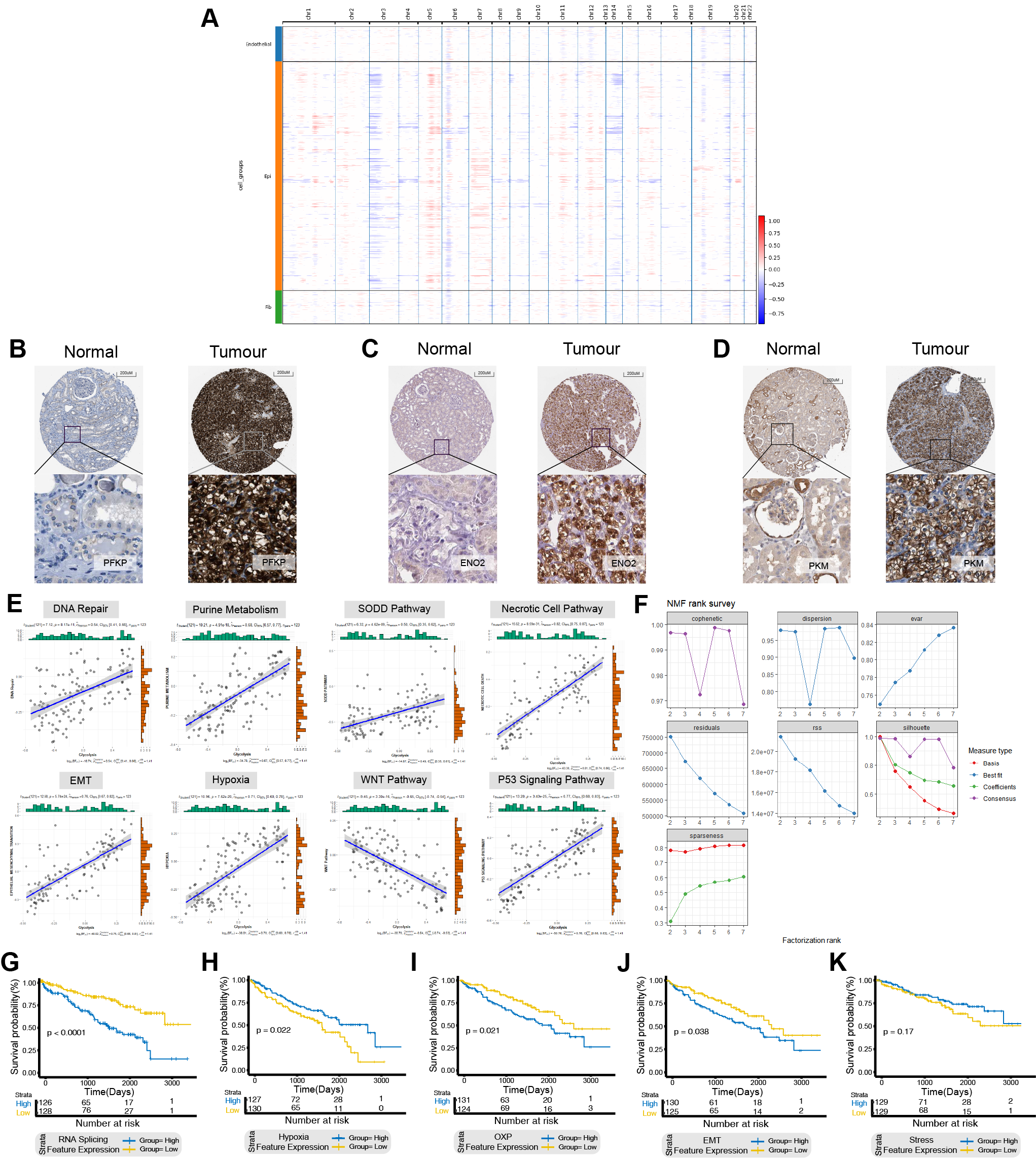

Supplement: Supplementary file 2 — Additional file 2: Figure S2. Correlation of tumor cell glycolysis with pathways and prognosis in clear cell renal cell carcinoma. A Copy number variation heatmap in epithelial cells. B–D Immunohistochemical staining images of PFKP/ENO2/PKM in adjacent normal tissue and clear cell renal cell carcinoma. E Scatter plots depicting the correlation of glycolytic activity with various biological features. F Stability plot of non-negative matrix factorization (NMF) clustering of tumor cells at different group numbers. G–K Survival analysis of tumor cells enriched in different groups based on RNA splicing, Hypoxia, Oxidative Phosphorylation (Oxp), Epithelial–Mesenchymal Transition (EMT), and Stress states. EMT: Epithelial–Mesenchymal Transition; Oxp: Oxidative Phosphorylation; NMF: Non-Negative Matrix Factorization. [file 12967_2024_4848_MOESM2_ESM.tiff]

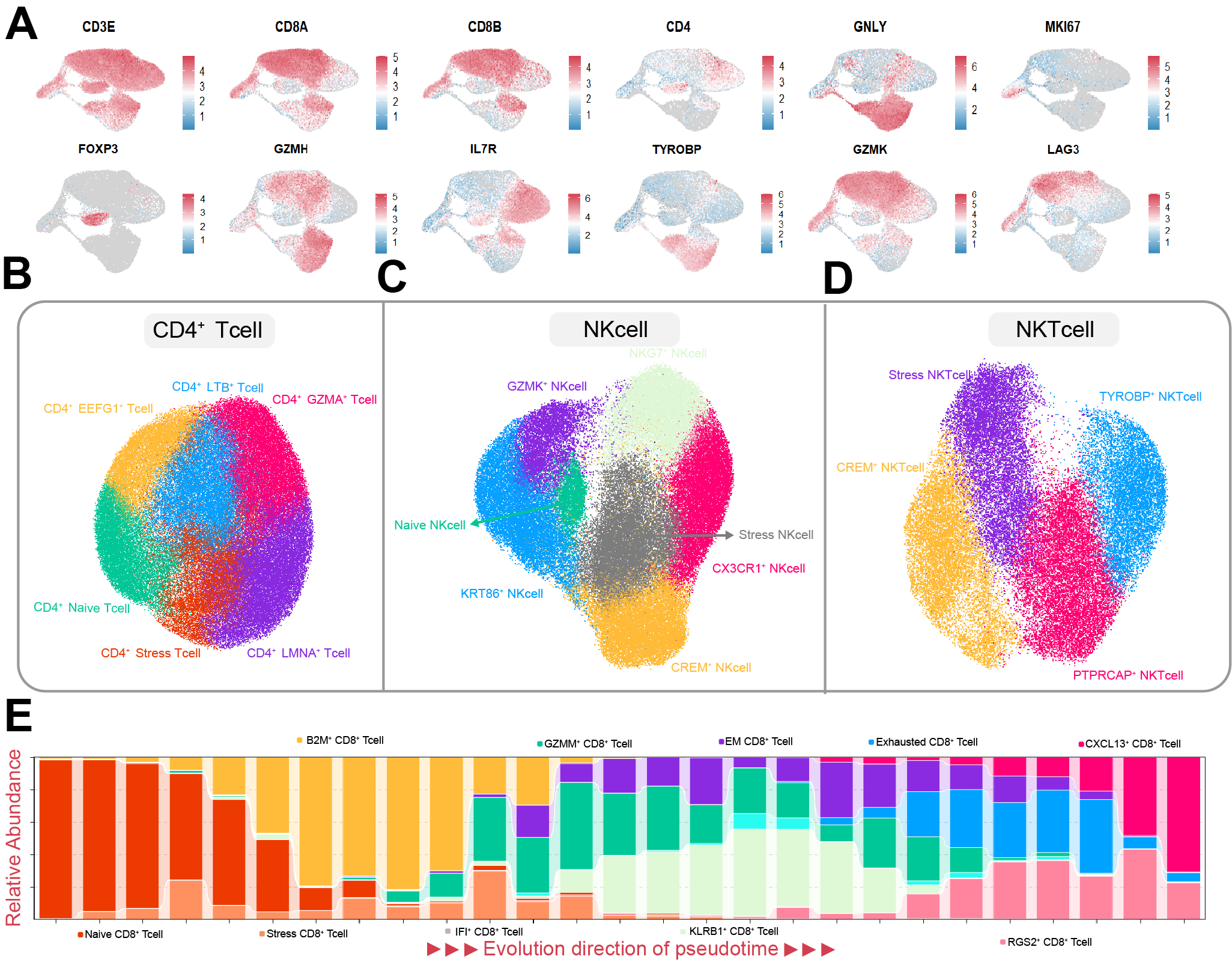

Supplement: Supplementary file 3 — Additional file 3: Figure S3. Metabolic dynamics of CD8+ T cells. A UMAP plot of T cells and NK cells, color-coded based on marker gene expression. B–D UMAP plots of CD4+ T cells, NK cells, and NKT cells, color-coded by cell subtype. E Dynamic changes in enrichment levels of different CD8+ T cell subtypes along the inferred pseudotime trajectory. UMAP: Uniform Manifold Approximation and Projection. [file 12967_2024_4848_MOESM3_ESM.tif]

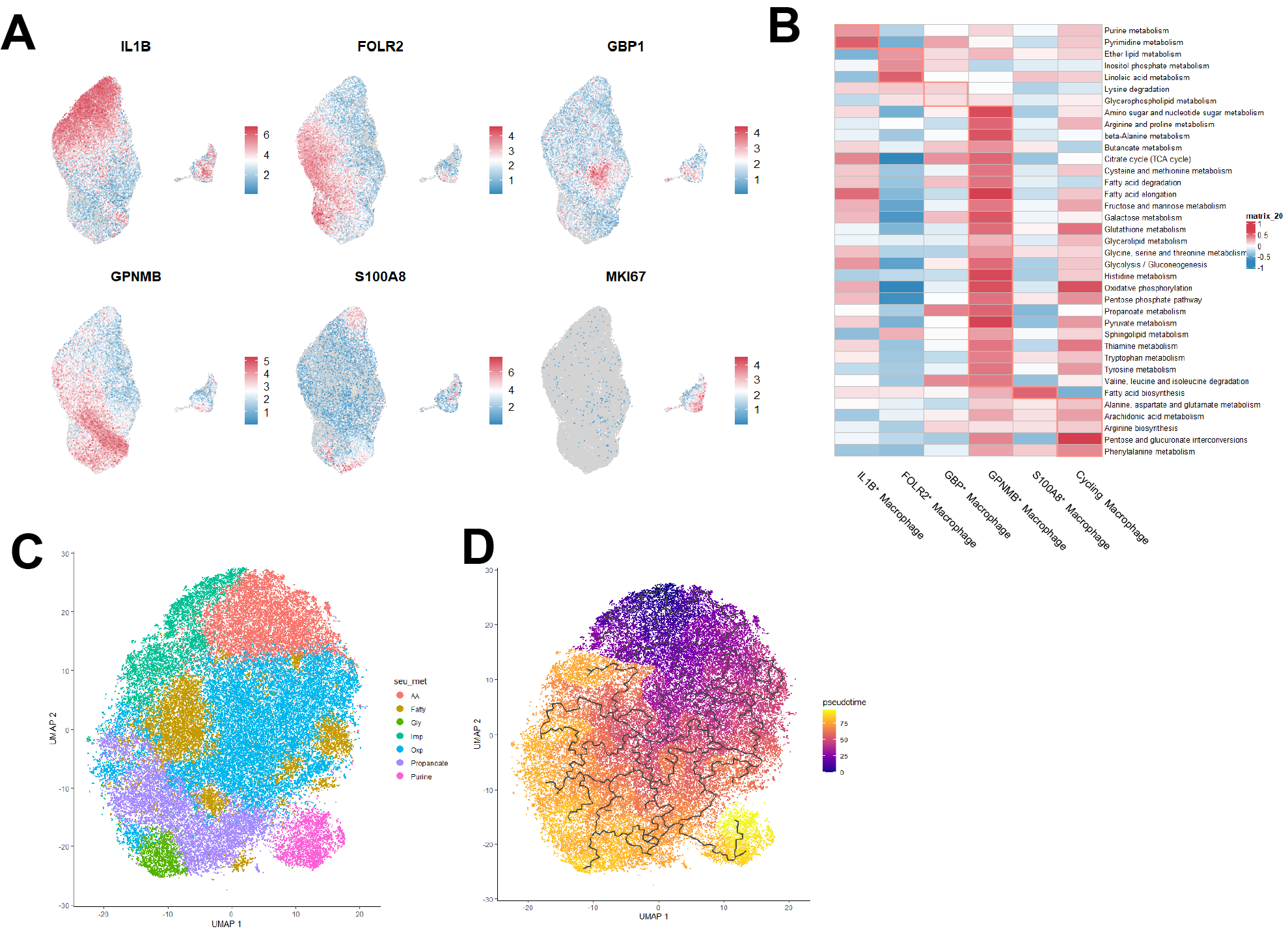

Supplement: Supplementary file 4 — Additional file 4: Figure S4. Dynamic evolution of macrophage metabolism. A UMAP plot of macrophages, color-coded by expression of marker genes. B Metabolic activity heatmap of macrophage subtypes across different cell subtypes. C UMAP plot of macrophages used for pseudotime analysis, color-coded by expression of marker genes. D UMAP plot of macrophages used for pseudotime analysis, color-coded by inferred pseudotime trajectory points, where black leans towards the starting point and yellow leans towards the endpoint of the trajectory. UMAP: Uniform Manifold Approximation and Projection. [file 12967_2024_4848_MOESM4_ESM.tif]

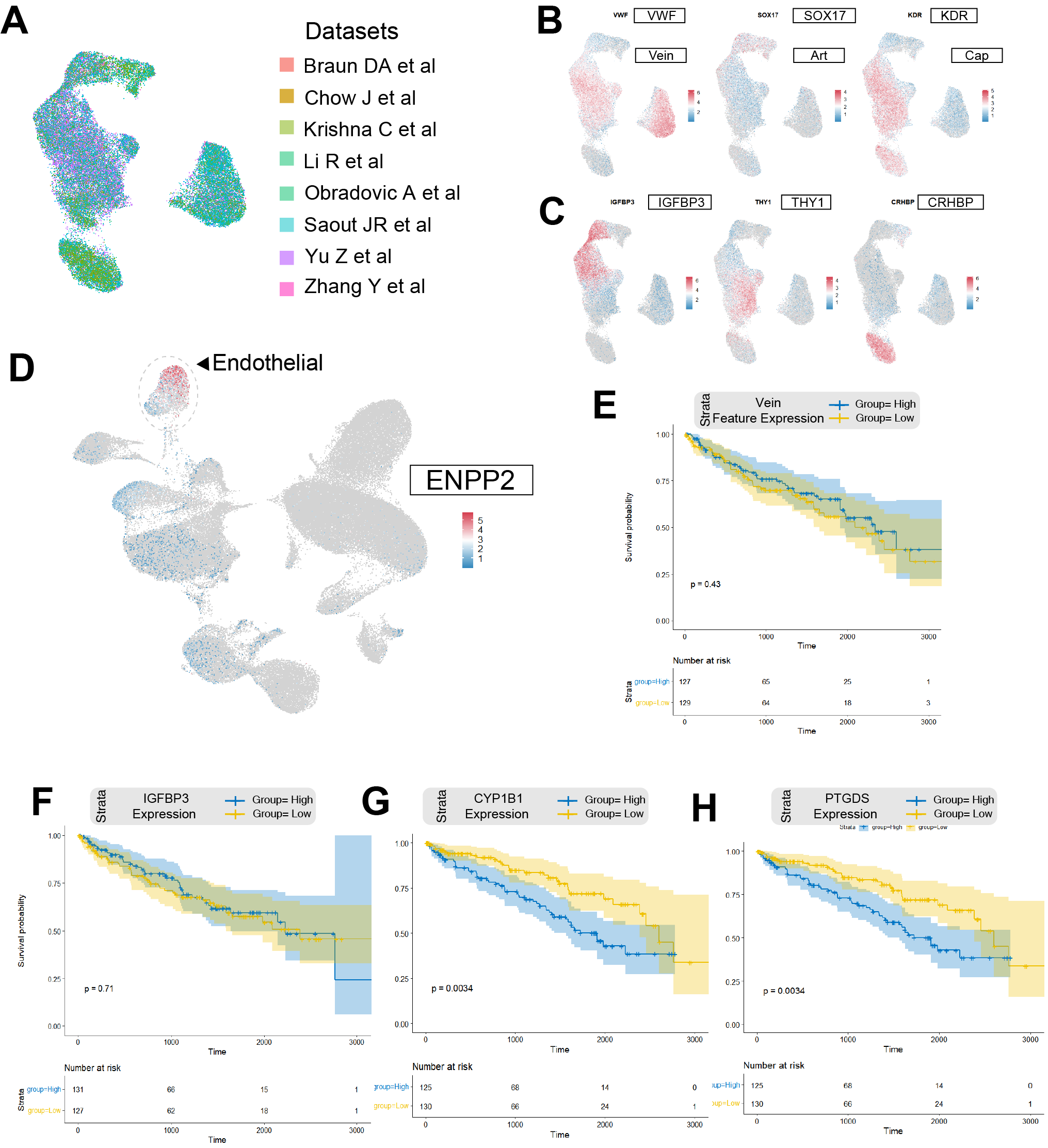

Supplement: Supplementary file 5 — Additional file 5: Figure S5. Correlation of tumor-originated endothelial cell-specific markers with prognosis. A UMAP plot of endothelial cells, color-coded based on their tissue of origin. B, C UMAP plots of endothelial cells, color-coded by the expression levels of specific marker genes. Red denotes high expression, blue denotes low expression. D UMAP plot displaying ENPP2 gene expression across all cells. Red indicates high expression, blue indicates low expression. E Prognostic analysis stratified by the expression of characteristic genes in venous endothelial cells. F–H Prognostic analysis stratified by the expression of IGFBP3, CYP1B1, and PTGDS genes. UMAP: Uniform Manifold Approximation and Projection. [file 12967_2024_4848_MOESM5_ESM.tif]

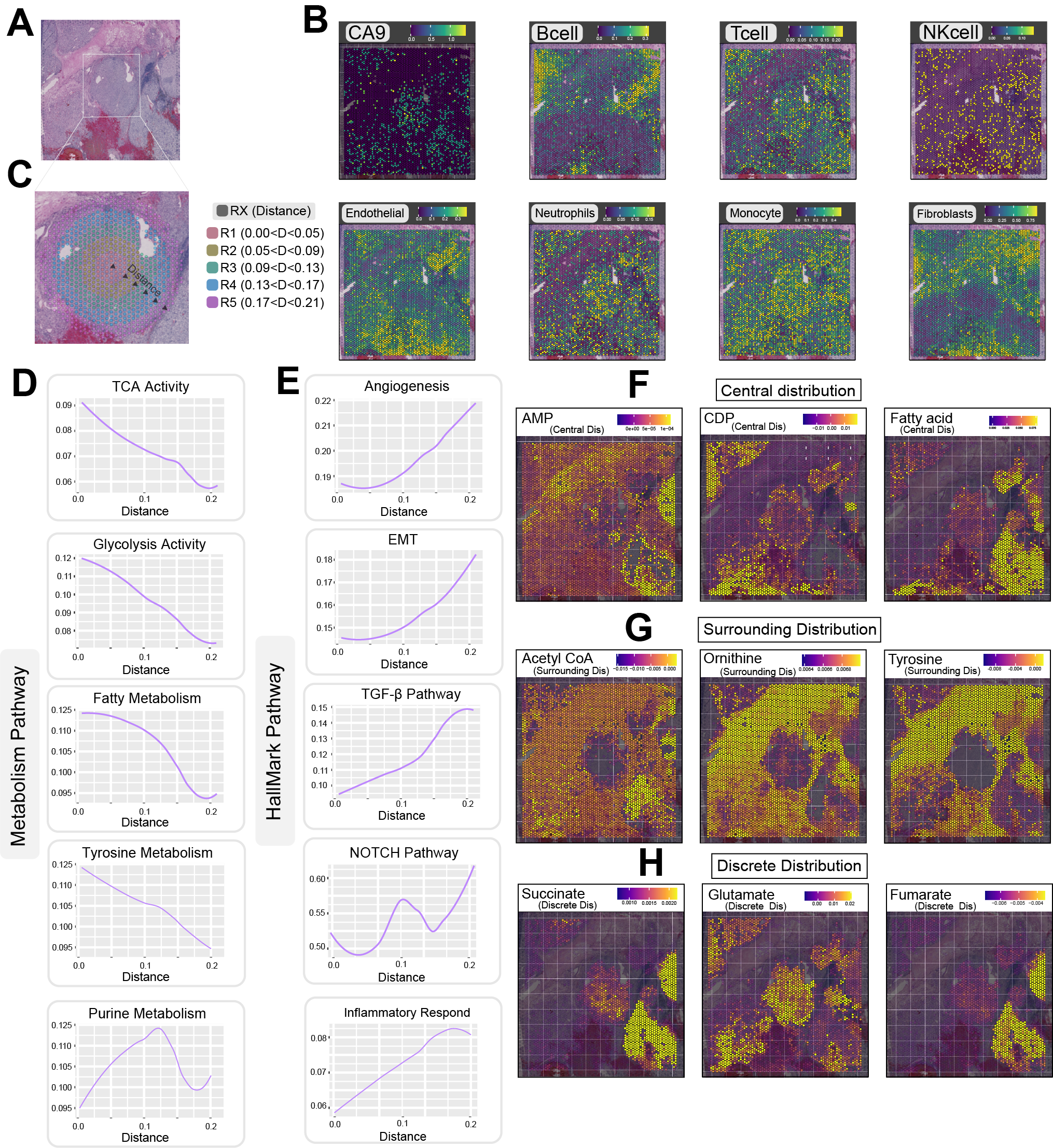

Supplement: Supplementary file 6 — Additional file 6: Figure S6. Slice 2: Heterogeneity in spatial metabolic activity. A Pathological section of spatial transcriptomics. B Approximate distribution of various cell types. C Partitioning of spatial transcriptomics data, colored by distance from the tumor center. D Correlation of metabolic pathways (glycolysis, TCA cycle, fatty acid metabolism, tyrosine, and purine metabolism) with distance from the tumor center. E Correlation of biological pathway activities (EMT, angiogenesis, inflammation, TGF-beta, and NOTCH) with distance from the tumor center. F Heatmap of balance flux for AMP, CDP, and fatty acids in spatial context. G Heatmap of balance flux for acetyl CoA, ornithine, and tyrosine in spatial context. H Heatmap of balance flux for succinate, glutamate, and fumarate in spatial context. EMT : Epithelial–Mesenchymal Transition. [file 12967_2024_4848_MOESM6_ESM.tif]

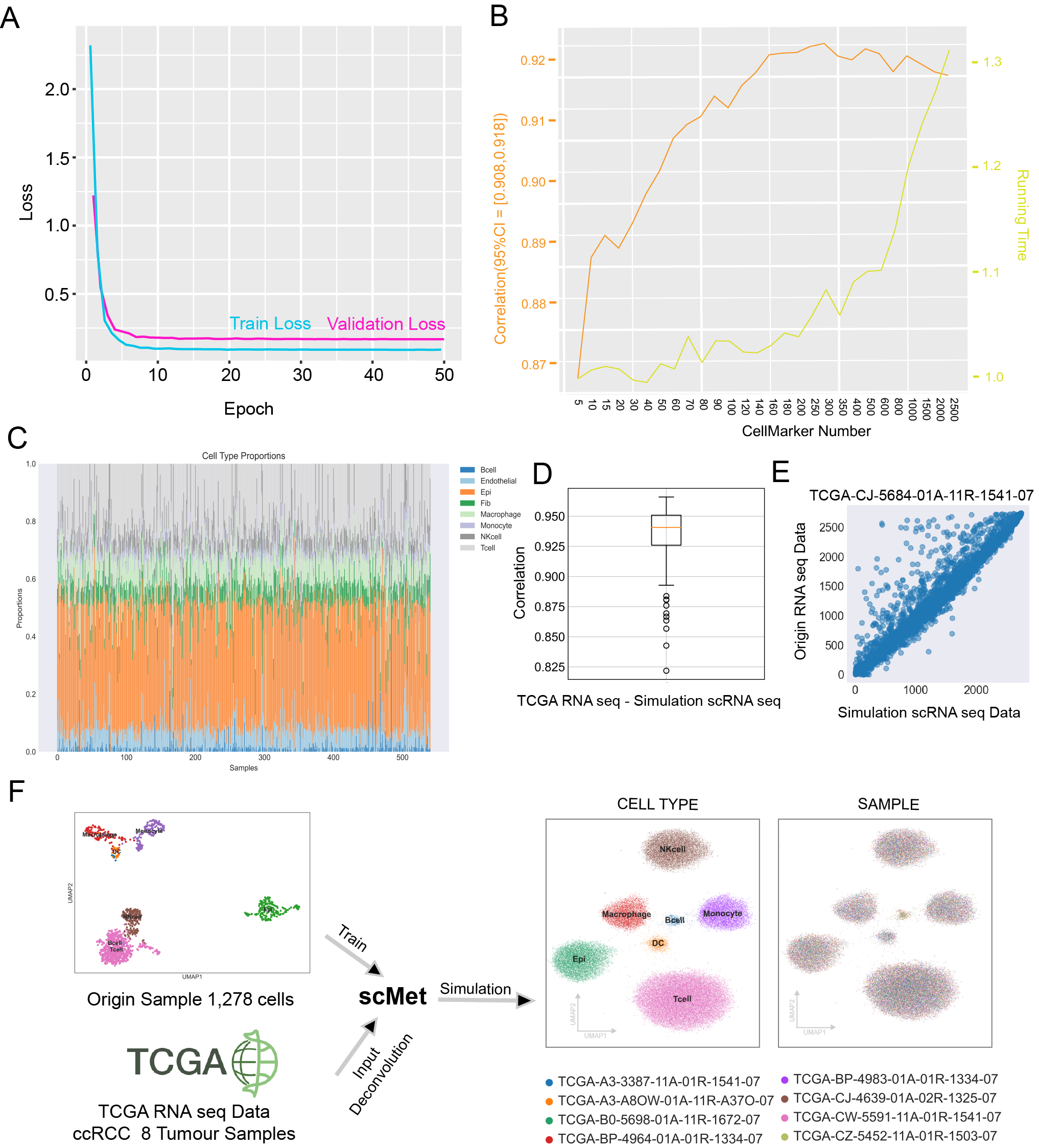

Supplement: Supplementary file 7 — Additional file 7: Figure S7. Figures for scMet program evaluation. A Correlation between the number of training iterations for the Conditional Variational Auto-Encoder (CVAE) model and the corresponding training loss and validation loss. B Correlation between the number of cell-type specific markers used for deconvolution of RNA sequencing data and the accuracy of computational results (Left), and correlation between the number of cell-type specific markers used for deconvolution of RNA sequencing data and computational time (Right). C Bar plot representing the cell type proportions obtained after deconvolution of TCGA RNA-seq data. D Line graph illustrating the gene expression correlation between the best-fitted scRNA-seq data and the original RNA-seq data. E Scatter plot depicting the correlation between gene expression of TCGA-CJ-5684-01A-11R-1541-07 RNA-seq data and the best-fitted scRNA-seq data. F Workflow illustrating the utilization of small sample scRNA-seq data to convert eight TCGA RNA-seq datasets into scRNA-seq data using scMet. CVAE: Conditional Variational Auto-Encoder. [file 12967_2024_4848_MOESM7_ESM.tiff]
