# Supplementary material for: Metabolic heterogeneity in clear cell renal cell carcinoma revealed by single-cell RNA sequencing and spatial transcriptomics
Source: J Transl Med. 2024 Feb 27;22:210. doi: 10.1186/s12967-024-04848-x (PMC10900752; doi:10.1186/s12967-024-04848-x)
Supplement: Supplementary file 11 — Additional file 11. Supplementary Materials And Methods. Expansion of the methodology section, details on software packages, and the operating platform used in the study. [file 12967_2024_4848_MOESM11_ESM.docx]

**Additional files**

**Materials and methods**

1. **Metabolic Differential Score Calculation:**

To quantify the expression differences of metabolism-related genes between tumor samples and adjacent normal samples in various cancers, RNA expression count matrices for multiple cancers were downloaded from the TCGA database (https://www.cancer.gov/ccg/research/genome-sequencing/tcga) and normalized using FPKM. The relevant code can be found in the "*Availability of data and material*" section.

The formula for Metabolic Differential Score Calculation:


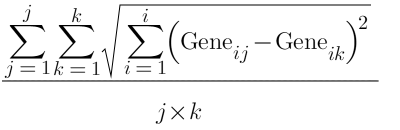


j: Number of tumor samples

k: Number of normal samples

i: Number of metabolic-related genes

Gene_ij_: Expression of the i-th metabolic-related gene in the j-th sample

Metabolic Differential Scores for each cancer are computed using the aforementioned formula. This formula aims to iterate over all combinations of normal and tumor tissues, calculating the expression differences for identical metabolism-related genes within each combination. The differences are squared and summed, and the average is obtained, resulting in a single Metabolic Differential Score for each cancer.

1. **Tumor Microenvironment Clustering**:

All samples annotated as tumors are included in the tumor microenvironment clustering based on cell proportions. On a per-sample basis, the proportions of cell subtypes, such as epithelial cells, endothelial cells, fibroblasts, monocytes, macrophages, dendritic cells, B cells, CD8+ T cells, CD4+ T cells, and proliferating T cells, are calculated. These proportions are then used as features for each sample.

Non-negative matrix factorization^1^ is employed for sample clustering. Initially, the clustering is performed without specifying the number of clusters. Subsequently, the number of clusters is set to range from 4 to 10, with each cluster count repeated 20 times. The final selection is based on higher cophenetic and dispersion values, with a preference for a larger cluster count, ultimately selecting 6 as the optimal number of clusters for tumor microenvironment classification. The extractFeatures function is utilized to extract key cell subtypes within each tumor microenvironment cluster.

1. **Calculating the Enrichment Level of Cell Types across Different Tissues:**

Using individual cell types and tissue types as calculation units, 2x2 contingency tables are generated based on cell annotation information. Fisher’s exact test is then applied to calculate P-values and Odds Ratios (ORs). Benjamini-Hochberg correction is utilized to adjust P-values. Odds Ratios close to 1 indicate independence, while values below or above 1 suggest negative or positive correlations, respectively. A smaller adjusted P-value implies a greater likelihood of differences in cell enrichment between tissues. Relevant code can be found at <https://github.com/gwyang6/scMet>.

Example:

To calculate the enrichment level of T cells in tumor tissues, a 2x2 contingency table is constructed as follows:

|  | T cells | Non-T cells |
| --- | --- | --- |
| Tumor Tissue | a | b |
| Non-Tumor Tissue | c | d |

A Fisher's exact test is applied to the 2x2 contingency table to obtain the Odds Ratio (OR) and P-value, where OR = ad/bc. Benjamini-Hochberg correction is then employed to adjust the P-value, reducing the probability of making erroneous rejections. The adjusted P-value is substituted for the original P-value.

Interpretation of results:

If the distribution of T cells in tumor tissues is similar to that in other tissues, showing no specific pattern, the calculated OR should be close to 1, and the obtained P-value should be greater than or equal to 0.05.

If T cells exhibit a significant enrichment in tumor tissues, the OR value should be greater than 1.5 or even 2, and the P-value should be much less than 0.05.

If T cells are relatively scarce in tumor tissues, the OR value should be less than 0.66 or even 0.5, and the P-value should be much less than 0.05.

This process is repeated for various cell types across different tissues, resulting in tables indicating the enrichment levels and corresponding P-values for each cell type in various tissues.

1. **Identification of Tumor Cells through Copy Number Variation:**

Copy number variations (CNVs) in epithelial cells were determined to distinguish between normal epithelial cells and tumor cells. InferCNVpy^2^ was employed for the calculation of CNVs in epithelial cells, with default parameters. As endothelial cells and fibroblasts exhibit relatively stable copy numbers, they were chosen as reference cells for CNV calculation. The copy number of each gene was squared after subtracting 1, and the sum was calculated for each cell, resulting in the total copy number variation score for each cell. In the epithelial cell population, if the copy number variation score for a particular cell was higher than that of endothelial cells and fibroblasts, it was classified as a tumor cell; otherwise, it was considered a normal epithelial cell.

1. **Tumor Cell Clustering and Feature Extraction:**

Due to significant inter-sample and intra-sample heterogeneity, the batch effect removal process often eliminates the heterogeneity of most tumor cells, resulting in the loss of features for heterogeneous tumor cells. Moreover, the limited number of highly variable genes may exclude certain genes with classification significance, such as those related to epithelial-mesenchymal transition. As a result, the conventional clustering analysis using Seurat^3^ and Harmony^4^ packages for epithelial cells yields results that are not sufficiently satisfactory.

Non-negative matrix factorization^1^ is employed for clustering all epithelial cells. To reduce computational complexity and retain tumor heterogeneity within samples, tumor cells from samples with more than 250 tumor cells are selected as input for the standard Seurat^3^ workflow. The FindClusters function, with the resolution parameter set to 0.3, is utilized for clustering tumor cells within each sample, and AverageExpression is employed to extract the average gene expression features for each class of tumor cells within the sample. After iterative processing for each sample, the results are merged, ultimately obtaining an expression matrix that preserves all gene information and intra-sample tumor heterogeneity.

The final expression matrix serves as input for non-negative matrix factorization^1^. Similar to the initial clustering, the first clustering is performed without specifying the number of clusters. Subsequently, the number of clusters is set to range from 4 to 10, with each cluster count repeated 20 times. The final selection is based on higher cophenetic and dispersion values, with a preference for a larger cluster count, ultimately selecting 5 as the optimal number of clusters. The extractFeatures function is utilized to extract key cell subtypes within each tumor microenvironment cluster.

1. **Survival Analysis:**

The required RNA expression matrix data and corresponding prognostic information for survival analysis are sourced from TCGA. Patient grouping is performed based on different features as outlined below:

1. Single-Gene Expression-based Grouping (e.g. ENPP2):

Samples are directly grouped based on the expression values of the gene count, such as *ENPP2*.

1. Cell Subtype-based Grouping:

The BayesPrism^5^ package is utilized for deconvolution to estimate the proportions of various cell subtypes within each sample. This method models the prior distribution based on cell type-specific expression profiles from scRNA-seq data. The deconvolution module is employed to jointly estimate the composition of cell types and the posterior distribution of cell type-specific gene expression in tumor (or non-tumor) samples with Bulk RNA-seq expression. Samples are grouped according to the target cell proportions.

1. Genesets Feature Score-based Grouping:

The Seurat^3^ package's FindMarkers function is used to identify feature genes for each cell subtype. The top 50 genes, ranked by fold change, are selected as feature genes for each subtype. If the number of genes with a fold change greater than 1.41 is less than 50, all genes with a fold change greater than 1.41 are retained as feature genes. GSVA^6^ is then applied to perform feature gene enrichment analysis on the RNA sequencing expression matrix of TCGA samples. The resulting genesets feature scores are used as the basis for sample grouping.

To analyze survival differences between different groups, the cox proportional hazards model implemented in the R package survival is utilized, correcting for the effects of other clinical factors including tumor stage, gender, and patient age. Survival curves are fitted using the Kaplan-Meier formula in the R package survival and visualized using the ggsurvplot function of the R package survminer.

**7. Gene Set Enrichment Analysis:**

Gene set enrichment analysis was performed using the "gsva" function from the GSVA^6^ package. The analysis was conducted with the GSVA mode, and the minimum gene set size was set to 5 genes. For calculating the activity of gene sets within individual cells, the AUCell^7^ package was employed with a parameter of aucMaxRank=0.10, while other parameters were left at default values. GO analysis was conducted using the "renrichGO" function from the ClusterProfiler^8^ package, focusing on biological processes (BP). The maximum p-value was set to 0.001, and the remaining parameters were kept as default.

**7. Pseudotime Trajectory Generation:**

The Monocle3 package^9^ is employed for pseudotime analysis of cell clusters, utilizing scRNA-seq objects generated through the Seurat^3^ standard workflow. This analysis aims to reveal the evolutionary sequence of cells. The UMAP or t-SNE representation used for pseudotime analysis is based on the Harmony^4^ and Seurat^3^ basic workflows. Default parameters are used for Monocle3 unless specifically stated otherwise. The selection of the starting point for the pseudotime sequence is guided by biological knowledge.

**8. Transcription Factor Analysis:**

The Count matrix of scRNA-seq data served as input for the pyscenic package.^10^ Transcription factor activity was calculated as described. To reduce computation time, we randomly selected 20,000 cells as representative data for calculating CD8^+^ T cell/Macrophage transcription factor activity. The calculated transcription factor activity for each cell was imported into R, and those with values exceeding a threshold were considered "active," while others were considered "inactive." The percentage of active transcription factors in each cell type was calculated.

1. **Metabolite Balance State Analysis:**

The scFEA^11^ package is employed to infer location-specific metabolic fluxes from spatial transcriptomics data. scFEA utilizes a multi-layer neural network to capture the intricate information cascade from the transcriptome to the metabolome, thereby overcoming the non-linear dependencies between enzyme gene expression and reaction rates. Using the Recon2.2 gene-metabolite network as a reference metabolic pathway, the model undergoes 100 repeated training sessions for each unit. The final outcome is the metabolic flux for each unit, where positive values indicate the accumulation of metabolites within the unit, and negative values signify the outflow of metabolites.

1. **The scMet program workflow:**

(1) Data Retrieval: Accessing gene expression data from multiple single-cell sequencing experiments along with their respective cell type annotation files. It is strongly recommended to leverage single-cell RNA sequencing data obtained from individual samples, rather than aggregating datasets with clear batch effects. The datasets are then merged into a unified sample, and cells are randomly selected to generate multiple simulated RNA-seq datasets.

1. Batch Effect Removal: Employing Combat^12^ for batch correction to mitigate technical variations between scRNA-seq data and Bulk RNA-seq data. This procedure yields batch-corrected Bulk RNA-seq data, subsequently utilized for deconvolution.

(3) Utilizing NNLS for Deconvolution to Estimate Cell Proportions in the Samples: Preprocessing of the scRNA-seq data (all single-cell data analysis during program execution and UMAP plot generation is performed using scanpy^13^). Marker genes and their expressions for each cell type are identified based on the corresponding cell annotation file. Subsequently, the NNLS (Non-Negative Least Squares) method is employed for deconvolution of the RNA-seq data, facilitating the determination of proportions for each cell type. NNLS is a technique used to solve linear systems of equations, with the unknown variables representing the cell proportions within the sample. In the linear system, the goal is to minimize the residual by finding the cell proportion vector y through the known cell-type-specific gene expression matrix A and the target RNA-seq data vector x for deconvolution.

(4) Training CVAE Model for Generating scRNA-seq Data:

Fitting RNA-seq data based on existing single-cell sequencing often encounters challenges such as cell duplication and insufficient cell numbers. Therefore, we leverage the advantages of Conditional Variational Autoencoder (CVAE) to learn the distribution pattern of the current data and generate a substantial volume of meaningful data for further analysis. By constructing a CVAE model and training it using single-cell sequencing data, we enable the model to comprehend the distribution pattern of the data. Additionally, we incorporate cell types as one of the training labels, allowing the model to understand the distinctions among various cell types.

The ultimately trained model is capable of generating a large amount of synthetic single-cell sequencing data, which can be utilized for subsequent fitting. The following provides a brief overview of the algorithm employed in the CVAE model:

The Conditional Variational Autoencoder (CVAE) model comprises three main components: an encoder function (μ, logσ^2 = Encoder(x, c)), a reparameterization step (z = μ ^+^ ε * exp(logσ/2) where ε ~ N(0, 1) element-wise), and a decoder function (x′ = Decoder(z, c)). The loss function (L(x, x′, μ, logσ^2)) is a combination of the Reconstruction Loss (measuring similarity between the input data x and the reconstructed data x') and the KL Divergence (quantifying the deviation of the distribution q(z|x, c) from the prior distribution p(z), which is usually assumed to be a standard normal distribution).

Annotation:

In the context of the CVAE model, the following variables are defined:

· x: Single-cell gene expression profiles.

· c: Cell types, provided as conditional information.

· z: Latent variables, which are sampled from the latent distribution during the reparameterization step.

· x′: New single-cell gene expression profiles (reconstructed data) generated by the decoder.

Goal: The objective of the CVAE model is to minimize the defined loss function by learning meaningful latent representations that effectively capture both the input data and the associated conditional information. The model can generate informative and structured latent representations for downstream analyses and applications.

1. Parameter Selection for Generating Realistic scRNA-seq Data Using the Trained CVAE Model:

The data generated by the trained CVAE model may not be inherently reasonable, necessitating user intervention to optimize the standard deviation for the generated scRNA-seq data. For varying user-input standard deviations, the model will produce small-scale scRNA-seq datasets, and UMAP plots will be generated following the Scanpy^13^ workflow to allow users to assess the rationality of the generated data. This iterative process continues until the most suitable parameters are selected for input into the next steps.

1. Generating Large-Scale scRNA-seq Data：Utilizing the trained CVAE model and the selected parameters, large-scale scRNA-seq data is generated. The quantity of data can reach millions or even tens of millions, providing a vast amount of single-cell expression data. This dataset will be employed in the subsequent fitting steps.
2. Fitting scRNA-seq Data to RNA-seq Data: Randomly selecting a specified number of scRNA-seq data points in each iteration, these are fitted to the RNA-seq data. The correlation and Euclidean distance are recorded for each fitting. Ultimately, the scRNA-seq data demonstrating the most robust correlation with the RNA-seq data is selected as the optimal representation.

The primary inputs for the program include scRNA-seq data and RNA-seq data. The main outcome is a refined set of scRNA-seq data, serving as an exemplary surrogate for RNA-seq data.

scMet is a Python-based program and is currently not compatible with the R language.

| Package/Platform | Version |
| --- | --- |
| R | 4.3.0 |
| Seurat | 4.3.0 |
| Harmony | 1.0.3 |
| monocle3 | 1.3.1 |
| clusterProfiler | 4.8.3 |
| GSVA | 1.48.3 |
| ggplot2 | 3.4.4 |
| AUCell | 1.22.0 |
| BiocManager | 1.30.22 |
| BayesPrism | 2.1.0 |
| ComplexHeatmap | 2.16.0 |
| devtools | 2.4.5 |
| MCPcounter | 1.2.0 |
| msigdbr | 7.5.1 |
| Scrublet | 0.2.3 |
| NMF | 0.26.0 |
| survival | 3.5-5.0 |
| surminer | 0.4.9 |
| patchwork | 1.1.3 |
| GSEABase | 1.62.0 |
| python | 3.9.12 |
| InferCNVpy | 0.3.0 |
| pySCENIC | 0.12.1 |
| scFEA | v1.1-beta0.1 |
| scMet | 0.1.2 |
| Combat | 0.3.3 |
| Scanpy | 1.9.6 |
| Pytorch | 2.1.0 |

| Platform 1 | Device Information |
| --- | --- |
| CPU | Intel(R) Xeon(R) CPU E5-2680 v4 @ 2.40GHz |
| GPU | NVIDIA GeForce RTX 3090 24GB *4 |
| Memory | 512G DDR4 3600MHz |

| Platform 2 | Device Information |
| --- | --- |
| CPU | Intel(R) Xeon(R) CPU E5-2678 v3 @ 2.50GHz |
| GPU | NVIDIA GeForce GTX 960 4G |
| Memory | 128G DDR3 1866MHz |

**References**

1. Lee DD, Seung HS. Learning the parts of objects by non-negative matrix factorization. Nature. 1999;401(6755):788-791. doi:10.1038/44565
2. icbi-lab. GitHub - icbi-lab/infercnvpy: Infer copy number variation (CNV) from scRNA-seq data. Plays nicely with Scanpy.
3. Hao Y, Hao S, Andersen-Nissen E, et al. Integrated analysis of multimodal single-cell data. Cell. 2021;184(13):3573-3587.e29. doi:10.1016/j.cell.2021.04.048
4. Korsunsky I, Millard N, Fan J, et al. Fast, sensitive and accurate integration of single-cell data with Harmony. Nat Methods. 2019;16(12):1289-1296. doi:10.1038/s41592-019-0619-0
5. Chu T, Wang Z, Pe'er D, Danko CG. Cell type and gene expression deconvolution with BayesPrism enables Bayesian integrative analysis across bulk and single-cell RNA sequencing in oncology. Nat Cancer. 2022;3(4):505-517. doi:10.1038/s43018-022-00356-3
6. Hänzelmann S, Castelo R, Guinney J. GSVA: gene set variation analysis for microarray and RNA-seq data. BMC Bioinformatics. 2013;14:7. Published 2013 Jan 16. doi:10.1186/1471-2105-14-7
7. Aibar S, González-Blas CB, Moerman T, et al. SCENIC: single-cell regulatory network inference and clustering. Nat Methods. 2017;14(11):1083-1086. doi:10.1038/nmeth.4463
8. Wu T, Hu E, Xu S, et al. clusterProfiler 4.0: A universal enrichment tool for interpreting omics data. Innovation (Camb). 2021;2(3):100141. Published 2021 Jul 1. doi:10.1016/j.xinn.2021.100141
9. Cao J, Spielmann M, Qiu X, et al. The single-cell transcriptional landscape of mammalian organogenesis. Nature. 2019;566(7745):496-502. doi:10.1038/s41586-019-0969-x
10. Van de Sande B, Flerin C, Davie K, et al. A scalable SCENIC workflow for single-cell gene regulatory network analysis. Nat Protoc. 2020;15(7):2247-2276. doi:10.1038/s41596-020-0336-2
11. Alghamdi N, Chang W, Dang P, et al. A graph neural network model to estimate cell-wise metabolic flux using single-cell RNA-seq data. Genome Res. 2021;31(10):1867-1884. doi:10.1101/gr.271205.120
12. Zhang Y, Parmigiani G, Johnson WE. ComBat-seq: batch effect adjustment for RNA-seq count data. NAR Genom Bioinform. 2020;2(3):lqaa078. doi:10.1093/nargab/lqaa078
13. Wolf FA, Angerer P, Theis FJ. SCANPY: large-scale single-cell gene expression data analysis. Genome Biol. 2018;19(1):15. Published 2018 Feb 6. doi:10.1186/s13059-017-1382-0
